# MiR-4638-5p inhibits castration resistance of prostate cancer through repressing Kidins220 expression and PI3K/AKT pathway activity

**DOI:** 10.18632/oncotarget.10165

**Published:** 2016-06-18

**Authors:** Yang Wang, Ning Shao, Xueying Mao, Minmin Zhu, Weifei Fan, Zhixiang Shen, Rong Xiao, Chuncai Wang, Wenping Bao, Xinyu Xu, Chun Yang, Jian Dong, Deshui Yu, Yan Wu, Caixia Zhu, Liting Wen, Xiaojie Lu, Yong-Jie Lu, Ninghan Feng

**Affiliations:** ^1^ Department of Urology, Affiliated Wuxi No. 2 Hospital of Nanjing Medical University, Wuxi, China; ^2^ Wuxi Medical School, Jiangnan University, Wuxi, China; ^3^ Centre for Molecular Oncology, Barts Cancer Institute, Queen Mary University of London, London, United Kingdom; ^4^ Jiangsu Province Geriatric Institute, Nanjing, China; ^5^ College of Clinical Medicine, Nanjing Medical University, Nanjing, China; ^6^ Centre for Translational Medicine, Affiliated Wuxi No. 2 Hospital of Nanjing Medical University, Wuxi, China

**Keywords:** castration resistant prostate cancer, miR-4638-5p, Kidins220, PI3K/AKT pathway, angiogenesis

## Abstract

MicroRNAs (miRNAs) are short, conserved segments of non-coding RNA which play a significant role in prostate cancer development and progression. To identify miRNAs associated with castration resistance, we performed miRNA microarray analysis comparing castration resistant prostate cancer (CRPC) with androgen dependent prostate cancer (ADPC). We identified common underexpression of miR-4638-5p in CRPC compared to ADPC samples, which were further confirmed by quantitative PCR analysis. The role of miR-4638-5p in prostate cancer androgen-independent growth has been demonstrated both *in vitro* and *in vivo*. We also identified *Kidins220* as a target gene directly regulated by miR-4638-5p and shRNA-mediated knockdown of *Kidins220* phenocopied miR-4638-5p restoration. Subsequently, we revealed that *Kidins220* activates PI3K/AKT pathway, which plays a key role in CRPC. Loss of miR- 4638-5p may lead to CRPC through the activity of Kidins220 and PI3K/AKT pathway. Furthermore, we found that miR-4638-5p, through regulating Kidins220 and the downstream activity of VEGF and PI3K/AKT pathway, influences prostate cancer progression via angiogenesis. The identification of miR-4638-5p down-regulation in CRPC and the understanding of the functional role of miR-4638-5p and its downstream genes/pathways have the potential to develop biomarkers for CRPC onset and to identify novel targets for novel forms of treatments of this lethal form of PCa.

## INTRODUCTION

Prostate cancer (PCa) is the commonest malignancy in males in the western world and accounts for approximately 10% of all male cancer-related deaths in the USA [1]. Androgen deprivation therapy (ADT) is an effective therapeutic option for advanced PCa but castration resistance invariably develops [2]. In this context, chemotherapy and other systemic therapies have limited efficacy [3]. An improved understanding of the molecular mechanisms of castration resistance can guide rational development of novel therapeutic strategies.

miRNAs play critical roles in the regulation of cell biological processes [4, 5] and are involved in the development and progression of various human malignancies including PCa [6–12]. In PCa, miRNAs have been reported to regulate cancer cell growth, survival and sensitivity to chemotherapy [6, 7, 9, 11, 13]. Although differential expression of certain miRNAs between androgen-dependent prostate cancer (ADPC) and castration-resistant prostate cancer (CRPC) clinical samples has been reported, each of those studies was performed on only a few CRPC clinical samples. The cellular functions of these differentially expressed miRNAs identified in the clinical samples were also only investigated generally for PCa cell growth instead of the development of castration resistance [14–16]. We aimed to identify novel miRNAs associated with castration resistance in PCa and to investigate the downstream pathways and molecular mechanisms. We found decreased expression of miR-4638-5p in CRPC and revealed functionally that miR-4638-5p inhibited the growth of androgen independent cancer cells and that the inhibition of miR-4638-5p promoted androgen-independent cell growth for initially androgen dependent cells. Subsequently we identified Kidins220, VEGF and the PI3K/AKT pathway as the downstream mediators of miR-4638-5p in suppressing CRPC development as well as PCa angiogenesis *in vitro* and in xenografted nude mice.

## RESULTS

### MiR-4638-5p is significantly down-regulated in CRPC compared to ADPC samples

**Figure 1:**
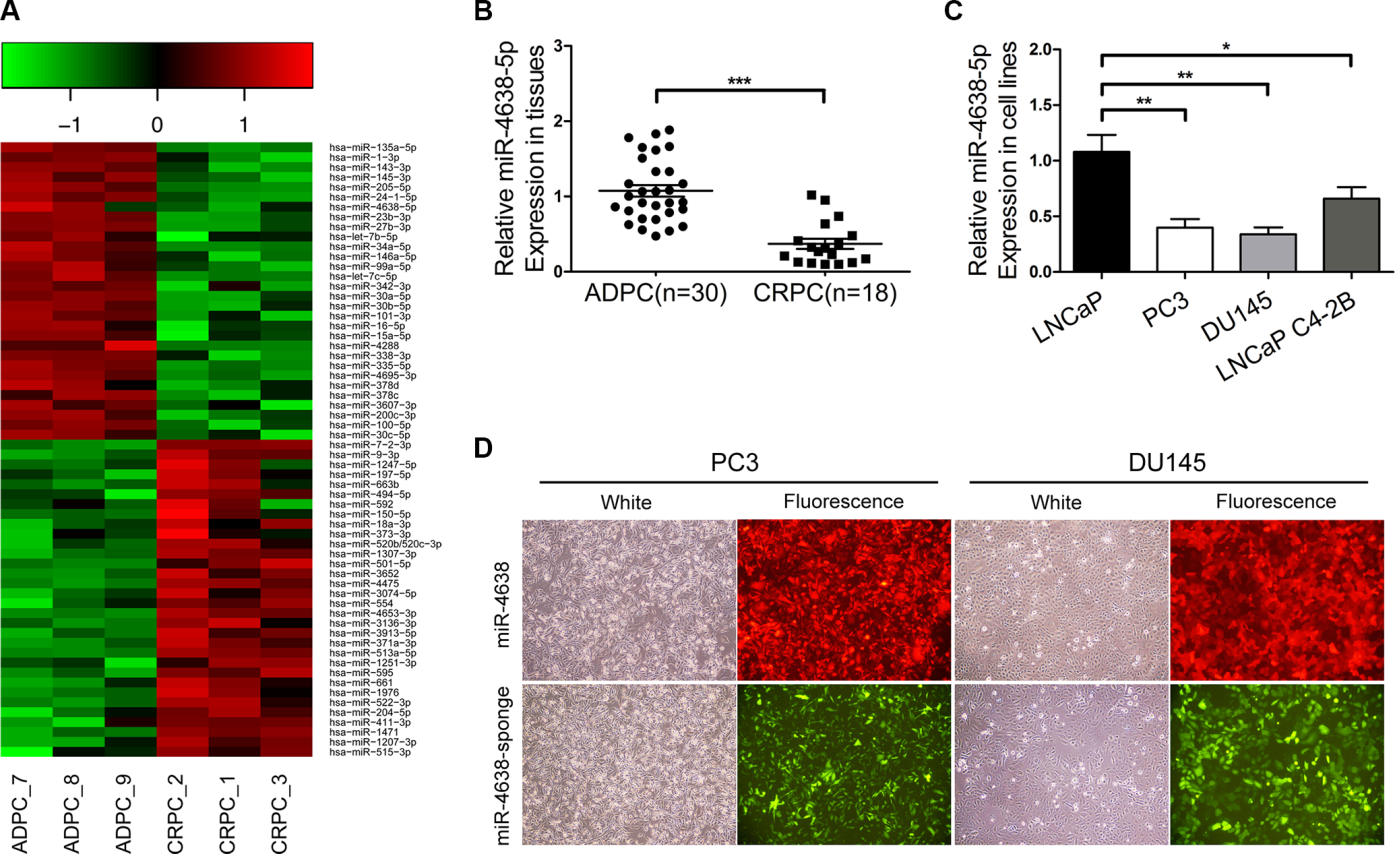
Downregulation of miR-4638-5p in CRPC and construction of PCa cell lines stably overexpressing/underexpressing miR-4638-5p (**A**) Heatmap of top miRNAs with differential expression in ADPC and CRPC tissues. Pseudo-color was used to represent intensity scale of miRNAs expression from the array analysis. Green and red denote low and high expression, respectively. (**B**) The expression levels of miR-4638-5p in 18 CRPC and 30 ADPC samples were detected by qRT-PCR. (**C**) The expression levels of miR-4638-5p in LNCaP, PC3, DU145 and LNCaP C4-2B cells were detected by qRT-PCR. * indicates *P* < 0.05, ** indicates *P* < 0.01 and *** indicates *P* < 0.001 by Student's *t*-test. (**D**) The expression of RFP and EGFP from the pCDH-miR-4638-5p (miR-4638) and the pCDH-miR-4638-5p sponge (miR-4638 sponge, to inhibit miR-4638-5p) were observed under the fluorescence microscope after that PCa cells were infected with lentivirus 72 hours under the same multiplicity of infection. (original magnification, ×100).

To explore the expression change of miRNAs associated with castration resistance, we performed miRNA microarray experiments using RNA samples extracted from three CRPC and three ADPC samples (Supplementary Table S1). We found 30 underexpressed (Supplementary Table S2) and 32 overexpressed miRNAs (Supplementary Table S3) with >2 fold difference of mean value in the three CRPC samples compared with the ADPC samples (Figure 1A). 25 underexpressed and 16 overexpressed miRNAs have been previously reported in human cancers, including PCa, such as underexpression of miR-135a [17, 18], miR-143 [17, 19], miR-145 [17, 20], miR-205 [17, 21–24], miR-24-1 [17, 25], miR-146a [26, 27], miR-3607 [28], and overexpression of miR-1247 [29], miR-197 [20], miR-592 [30], miR-150 [31, 32]. Among the 30 underexpressed miRNAs in CRPC samples, miR-4638-5p, a miRNA that has not been previously reported in any human cancers, was expressed 2.4-fold higher in the ADPC than CRPC samples. Although it has not been reported in a previous publication [33], we found that miR-4638-5p was expressed 4.7-fold higher in early-stage compared to advanced disease in the miRNA microarray data from that study. In a separate miRNA microarray analysis of PCa stem cells, which are closely associated with CRPC development [34], miR-4638- 5p was the most significantly down-regulated miRNA compared with the unselected population of DU145 cells, with 12.6-fold difference (unpublished data). All these data support that reduced expression of miR-4638-5p may be responsible for a more aggressive phenotype. We further examined its expression in 30 ADPC tissues and 18 CRPC tissues by qRT-PCR. In addition to confirming the CRPC-associated downregulation of miR-4638-5p in the six samples used for microarray analysis, we found that miR-4638-5p expression level was significantly lower in CRPC compared to ADPC samples (*P* = 1.44 × 10^−8^) (Figure 1B) in this cohort of samples. We also determined the expression levels of miR-4638-5p in LNCaP, PC3, DU145 and LNCaP C4-2B PCa cell lines, consistent with our observation in the clinical samples, miR-4638- 5p expression at a much higher level in LNCaP androgen sensitive cells than the three androgen-independent PCa cell lines (Figure 1C).

### MiR-4638-5p suppresses androgen-independent PCa cell growth *in vitro* and *in vivo*

**Figure 2:**
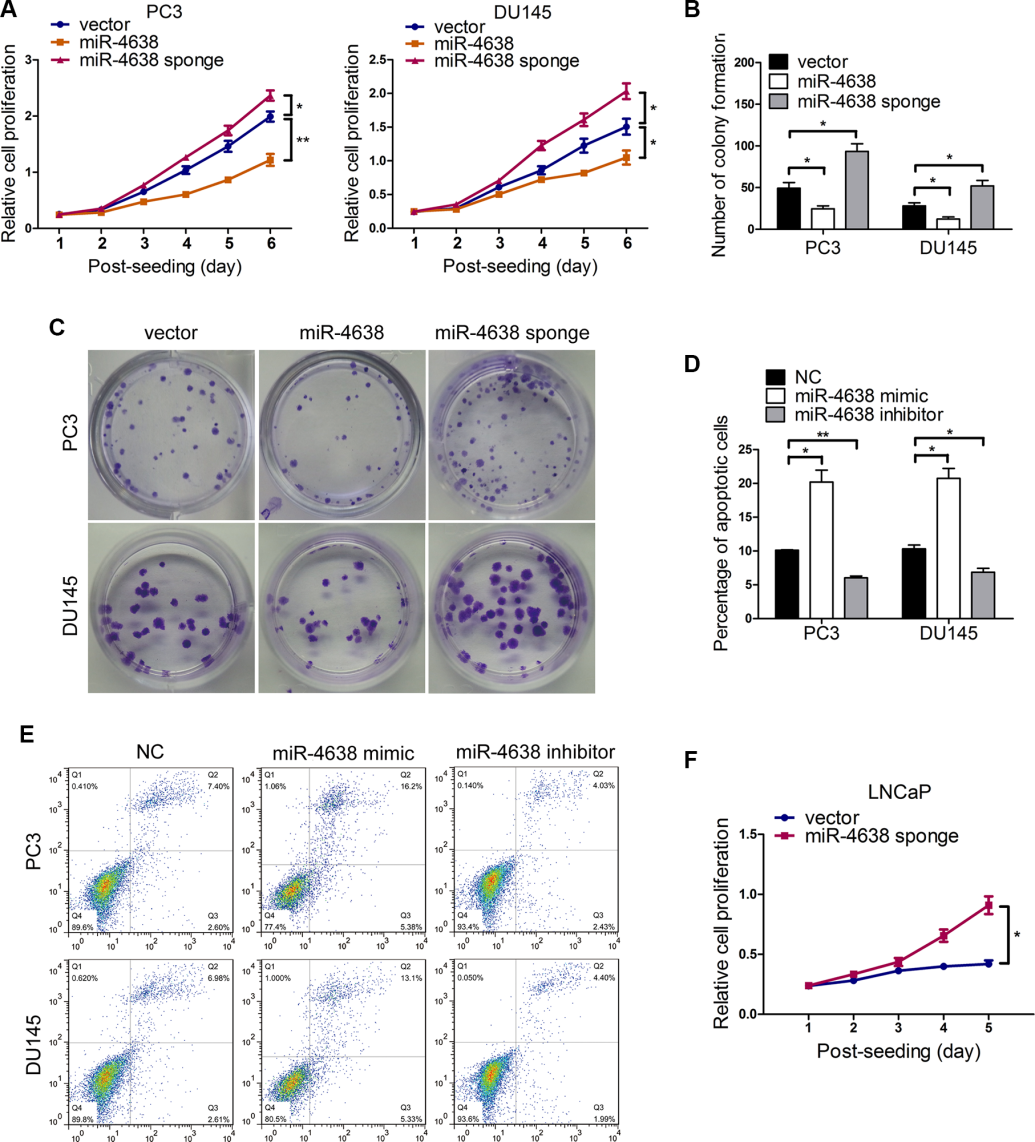
The effects of miR-4638-5p on cell proliferation, colony formation, apoptosis and the development of castration resistance (**A**) Cell proliferation results of PC3 and DU145 cells transduced with the pCDH-miR-4638-5p (miR-4638) or the pCDH-miR-4638-5p sponge (miR-4638 sponge, to inhibit miR-4638-5p), determined by a Cell Counting Kit-8 assay (CCK-8). Data represent mean ± SD determined from three independent experiments (*n* = 3), each with four technical replicates. Representative images from CCK-8 assay, showing the reduced or increased proliferative capacity of both PC3 and DU145 cells after stably overexpressing miR- 4638-5p or miR-4638-5p sponge, respectively, in comparison with cells transduced with the pCDH-vector controls (vector). (**B**) Colony formation ability of PC3 and DU145 cells transduced with the pCDH-miR-4638-5p (miR-4638) or the pCDH-miR-4638-5p (miR-4638 sponge). Bars represent the mean ± SD from three independent experiments (*n* = 3), each with three technical replicates. (**C**) Representative images showing the increased or decreased colony formation ability of both PC3 and DU145 cells after stably overexpressing miR-4638-5p or miR-4638-5p sponge, respectively, in comparison with cells transduced with the pCDH-vector controls (vector) after 2 weeks of seeding (original magnification, ×100). (**D**) Cell apoptosis assay was performed by flow cytometry analysis. The forced expression of miR-4638-5p (miR-4638 mimic) significantly increased the sensitivity of PC3 and DU145 cells to apoptosis, compared with the scramble RNA negative control (NC). The percentage of total apoptotic cells decreased in response to miR-4638-5p inhibitor (miR-4638 inhibitor) transfection. Bars represent the mean ± SD from three independent experiments (*n* = 3), each with three technical replicates. (**E**) Representative FACS analysis images. (**F**) LNCaP cells stably expressing miR-4638-5p sponge proliferate significantly faster than the pCDH-vector control cells in androgen stripped cell culture medium. Bars represent the mean ± SD from three independent experiments (*n* = 3), each with four technical replicates. * indicates *P* < 0.05 and ** indicates *P* < 0.01 by Student's *t*-test.

We then explored the potential biological functions of miR-4638-5p in cancer cell growth in relation to androgen independence. We constructed androgen independent PC3 and DU145 PCa cell lines stably overexpressing or underexpressing miR-4638-5p (with pCDH-miR-4638-5p sponge) (Figure 1D). As expected, miR-4638-5p expression were increased by approximately 25- and 18-fold respectively in PC3 and DU145 cells transfected with pCDH-miR-4638-5p or reduced by 5- and 3-fold, respectively, in PC3 and DU145 cells transfected with pCDH-miR-4638-5p sponge, compared with each cell line transfected with empty-vector control (all *P* < 0.05) (Supplementary Figure S1). In addition, miR-4638- 5p evidently inhibited the reporter activity of pGL3-miR-4638-5p sensor reporter, further indicating that the miR-4638-5p lentiviral expression constructs in PCa cells were functional (Supplementary Figure S2). Overexpressing miR-4638-5p significantly reduced cell growth rate in comparison with cells transduced with the empty pCDH-vector control (*P* = 5.56 × 10^−3^ and *P* = 4.42 × 10^−2^for PC3 and DU145 cells, respectively) (Figure 2A), while the growth rate of PC3 and DU145 cells stably expressing miR-4638-5p sponge was significantly enhanced compared to cells transduced with the pCDH-vector control (*P* = 4.34 × 10^−2^ and *P* = 3.37 × 10^−2^ for PC3 and DU145 cells, respectively) (Figure 2A) as detected by CCK-8 assay. We also performed colony formation assay using those transfected cells and found that miR-4638-5p expressing PC3 and DU145 cells had lower colony formation abilities than cells transduced with the pCDH-vector control (*P* = 4.75 × 10^−2^ and *P* = 3.27 × 10^−2^ for PC3 and DU145 cells, respectively) and these cells stably expressing miR-4638-5p sponge showed opposite findings (*P* = 2.18 × 10^−2^ and *P* = 4.62 × 10^−2^ for PC3 and DU145 cells, respectively) (Figures 2B and 2C). To further explore whether the decline in cell growth rate of the above miR-4638-5p overexpressing CRPC cells is caused by the induction of apoptosis in addition to the reduced proliferative capacity, we analyzed the proportion of apoptotic cell by flow cytometry. The results demonstrated that the percentage of total apoptotic cells were significantly increased after miR-4638 mimic transfection in comparison to the scramble RNA negative control (*P* = 2.93 × 10^−2^ and *P* = 1.14 × 10^−2^ for PC3 and DU145 cells, respectively). In addition, PC3 and DU145 cells transfected with miR-4638 inhibitor significantly reduced cell apoptosis compared with the cells transfected with the scramble RNA negative control (*P* = 2.68 × 10^−3^ and *P* = 1.27 × 10^−2^, respectively) and miR-4638 mimic (Figure 2D and 2E). Subsequently, we investigated the effect on cell proliferation by reducing miR-4638- 5p expression level in androgen sensitive LNCaP cells. LNCaP cells cultured in non/very low androgen medium with charcoal-stripped serum have very limited proliferation capacity. However, when miR-4638-5p was inhibited by stably expressing miR-4638-5p sponge, LNCaP cells continuously proliferate and at day 5 there were significantly (*P* = 1.24 × 10^−2^) more cells than LNCaP transfected with the pCDH-vector control in androgen-stripped cell culture medium (Figure 2F), indicating that miR-4638-5p inhibits certain cellular pathways which are required for PCa cell proliferation when androgen is not available.

**Figure 3:**
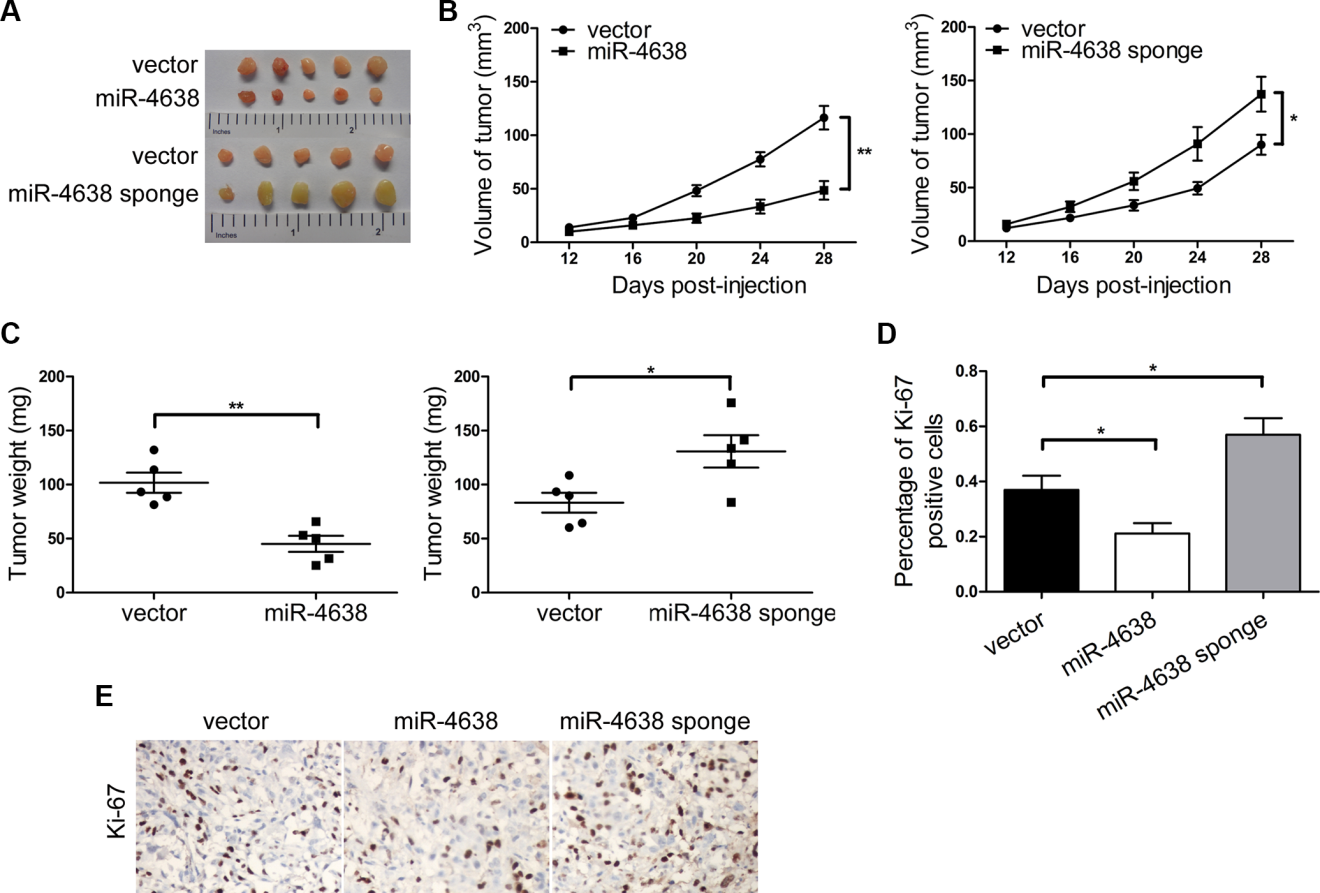
miR-4638-5p inhibits tumor growth in nude mice (**A**) Tumors grew in nude mice removed at day 28 after xenograft. (**B**) Overexpression of miR-4638-5p inhibited tumor growth and inhibition of miR-4638-5p using miR-4638-5p sponge promoted tumor growth of xenograpted PC3 cells based on two-dimensional caliper measurements of the tumor size. Data represent mean ± SD, each group with five tumours (*n* = 5). Two independent experiments were performed and similar results were obtained. (**C**) Overexpression of miR- 4638- 5p inhibited tumor growth and inhibition of miR-4638-5p using miR-4638-5p sponge promoted tumor growth of xenograpted PC3 cells based on the weight of those removed tumours. (**D**) miR-4638-5p overexpression and inhibition significantly decreased (*P* = 3.96 × 10^−2^) and increased (*P* = 3.51 × 10^−2^), respectively, Ki-67 expression detected by immunostaining in tumours from xonografted PC3 cells. (E) Representative immunostaining images (original magnification, ×100). * indicates *P* < 0.05 and ** indicates *P* < 0.01 by Student's *t*-test, respectively.

We then investigated the impact of miR-4638- 5p expression change on androgen independent tumor growth *in vivo*. We injected the PC3 cells overexpressing miR- 4638-5p into nude mice and observed a delay in tumor onset and a marked decrease in tumor growth when compared with the pCDH-vector control transfected cells that both the tumor volume and weight were significantly different at day 28 (*P* = 1.48 × 10^−3^ and *P* = 1.60 × 10^−3^, respectively). The opposite result was observed in cells transfected with pCDH-miR-4638-5p sponge compared to the pCDH-vector control (*P* = 4.36 × 10^−2^ and 3.23 × 10^−2^ for tumor volume and weight respectively) (Figures 3A–3C). In addition, Ki-67 positive cells were significantly reduced in PC3 cells transduced with pCDH-miR-4638-5p (*P* = 3.96 × 10^− 2^) and opposite result was observed in the PC3 cells transduced with pCDH-miR-4638-5p sponge (*P* = 3.51 × 10^−2^) (Figure 3D and 3E), suggesting that miR-4638-5p reduced tumor growth in the nude mouse model through inhibiting cell proliferation.

### MiR-4638-5p directly targets Kidins220

**Figure 4:**
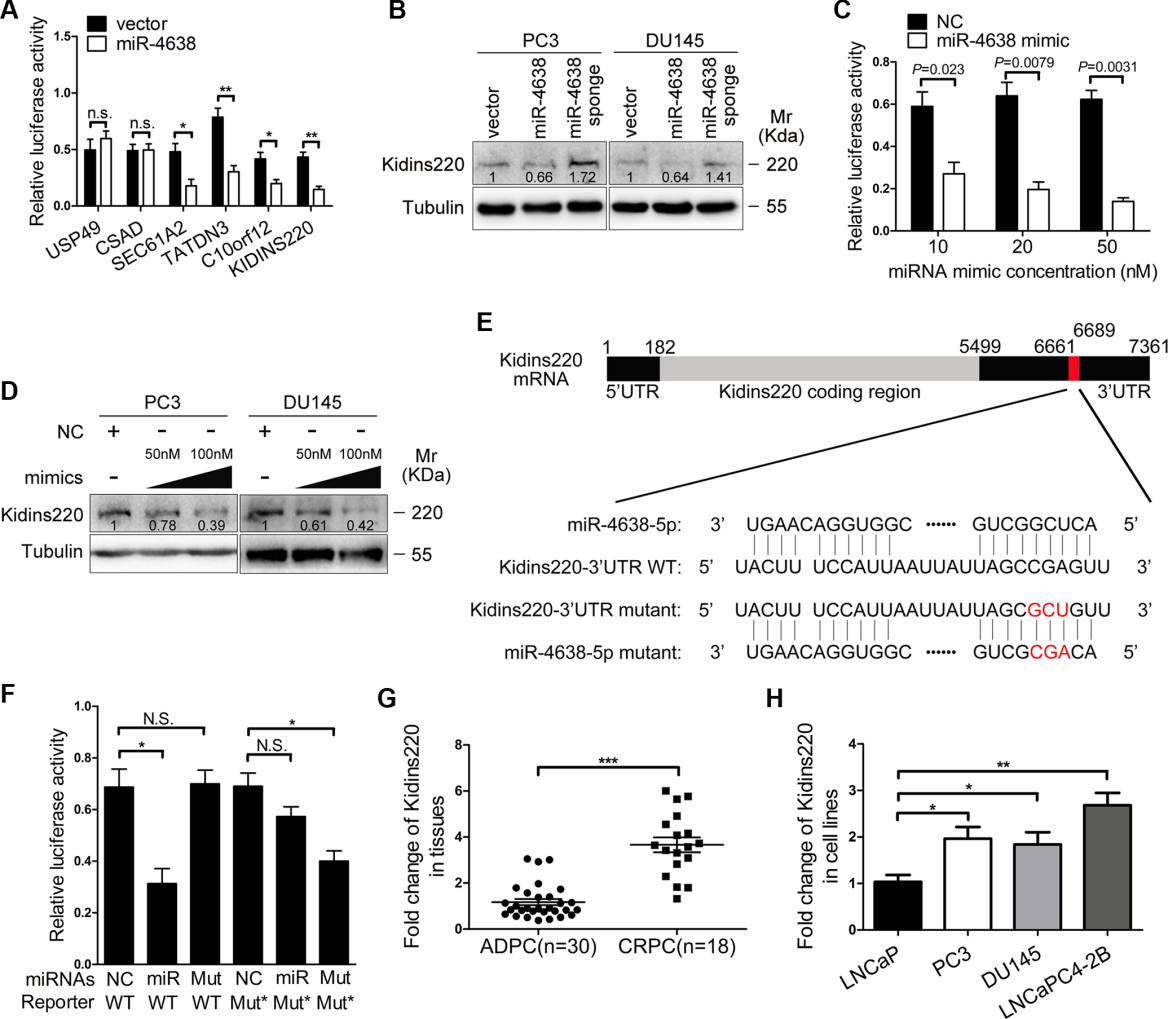
The regulation of Kidins220 by miR-4638-5p and the association of Kidins220 expression with CRPC (**A**) Luciferase activity under the control of 3′UTR of candidate genes in HEK293T cells transfected with the pCDH-miR-4638-5p (miR-4638) and the pCDH-vector control (vector). (**B**) The expression of Kidins220 protein in PC3 and DU145 cells transduced with the pCDH-miR-4638-5p (miR-4638) or the pCDH-miR-4638-5p sponge (miR-4638 sponge). (**C**) Luciferase activity under the control of *Kidins220* 3′UTR in HEK293T cells transfected with increasing amounts (10, 20 and 50 nM) of miR-4638 mimics for 48 hours. NC: scramble RNA negative control. (**D**) The expression of Kidins220 protein in PC3 and DU145 cells transfected with increasing amounts (50 and 100 nM) of miR-4638 mimics for 72 hour. (**E**) Schematic illustration of the putative seed sequence of miR-4638-5p within *Kidins220* 3′UTR and mutagenesis of binding site in the 3′UTR of *Kidins220* or miR-4638 mimic. (**F**) Effect of seed mutagenesis or mutation of the putative binding site on the *Kidins220* 3′UTR activity. Kidins220 wild type (WT) and mutant 3′UTR construct (Mut*) were co-transfected with the scramble RNA negative control (NC), natural (miR) or mutant miR-4638 mimic (Mut) into HEK293T cells. After co-transfection for 48 h, HEK293T cells were assayed for luciferase activity. In (A) (C) (F), the data represent the mean ± SD from three independent experiments (*n* = 3), each experiment containing four technical replicates. (**G**) *Kidins220* expression in 18 CRPC and 30 ADPC samples quantitated by qRT-PCR. (**H**) *Kidins220* expression in LNCaP, PC3, Du145 and LNCaP C4-2B cells. Relative quantities of mRNA expression are represented on the y-axis. *, ** and *** indicate *P* < 0.05, *P* < 0.01 and *P* < 0.001 respectively. n.s.: not significant.

Since miRNAs usually inhibit the translation of proteins via non-perfect pairing of six to eight nucleotides in length [35] or induce the degradation of target mRNAs in the case of perfect complementarity with the potential binding sites of target genes [36], bioinformatics analysis with several programs including TargetScan, RNAhybrid, Findtar, and Pita, was performed to predict the putative miR-4638-5p targets. Based on non-perfect complementarity with the seed sequences of miR-4638-5p, some putative binding sites were predicted in the 3′UTRs of six potential target genes, *USP49*, *CSAD*, *SEC61A2*, *TATDN3*, *C10orf12* and *Kidins220*. Subsequent luciferase reporter assay using 3′-UTR region of the six candidate genes confirmed that the promoter activity of four out of the six genes were significantly inhibited by miR-4638-5p (*P* < 0.05 for all) (Figure 4A) in HEK293T cells. However, only Kidins220 protein expression was significantly inhibited by miR-4638-5p in PC3 and DU145 cells as determined by Western blotting analysis (Figure 4B), while the expression changes of the other three proteins were not significant. As expected, knockdown of miR-4638-5p increased the expression of Kidins220 protein in those cells (Figure 4B). Moreover, luciferase reporter assay showed that miR-4638-5p inhibited the activity of *Kidins220* 3′-UTR WT in a dose-dependent fashion in HEK293T cells (Figure 4C). As expected, overexpression of miR-4638-5p using 50 nM and 100 nM miR-4638 mimics also suppressed Kidins220 protein expression in PC3 and DU145 cells in a dose-dependent manner (Figure 4D), further supporting that *Kidins220* is the gene regulated by miR-4638-5p. To confirm the specificity of miR-4638-5p in targeting *Kidins220*, we performed 3′-UTR mutagenesis analysis, where we mutated the putative miR-4638-5p binding site in the *Kidins220* 3′-UTR WT (Figure 4E) and found that the mutant mimic lacking the seed sequences reduced the inhibitory effect of miR-4638-5p on luciferase expression (*P* < 0.05; Figure 4F). In addition, the mutant miR-4638 mimic designed to match the *Kidins2*20 3′-UTR mut (Figure 4E) exhibited a strong inhibitory effect on the mutant reporter (*P* = 1.28 × 10^− 2^; Figure 4F). These results indicated that miR-4638-5p directly targets *Kidins220* with specific binding sites at the seed sequences of 3′- UTR.

### MiR-4638-5p may suppress CRPC cell growth by inhibiting Kidins220 expression

**Figure 5:**
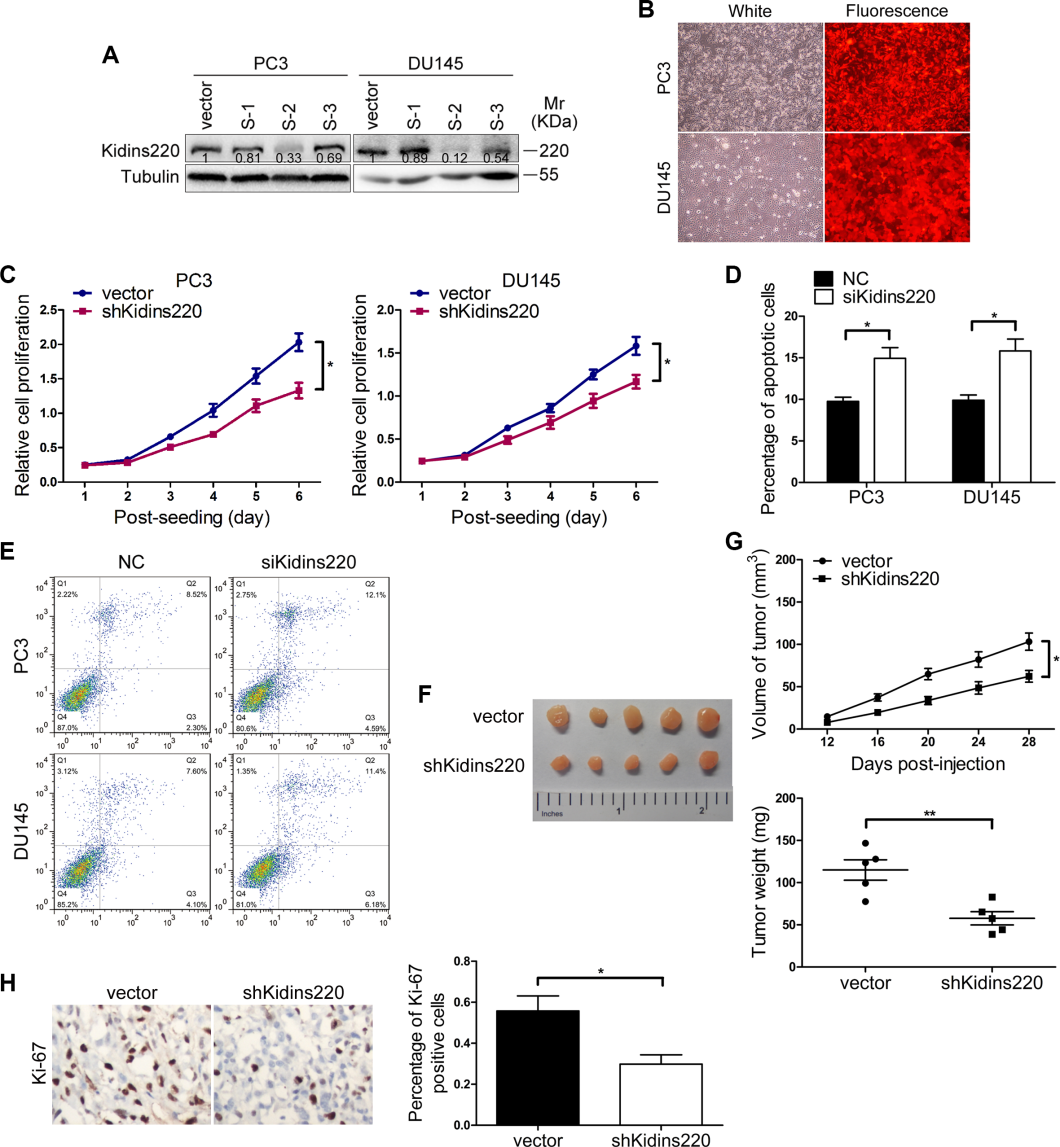
*Kidins220* knockdown mimics the effort of miR-4638-5p overexpression on tumor cell growth (**A**) Western blot analysis of Kidins220 expression levels of PC3 and DU145 cells transduced with shRNA1-3 (S1-3). (**B**) The expression of RFP were observed under the fluorescence microscope 72 hours after PC3 and DU145 cells were infected with pCDH-sh*Kidins220* (shKidins220) or the pCDH-vector controls (vector) under the same multiplicity of infection. (original magnification, ×100). (**C**) Cell proliferation results of PC3 and DU145 cells transduced with the pCDH-sh*Kidins220* (shKidins220) or the pCDH-vector controls (vector), determined by a Cell Counting Kit-8 (CCK-8) assay. Data represent mean ± SD determined from three independent experiments (*n* = 3), each with four technical replicates. Representative images from CCK-8 assay, showing the reduced proliferative capacity of both PC3 and DU145 cells after stably knockdown of *Kidins220*, in comparison with cells transduced with the pCDH-vector controls. (**D**) Cell apoptosis assay was performed by flow cytometry analysis. The knockdown of *Kidins220* (siKidins220) significantly increased the sensitivity of PC3 and DU145 cells to apoptosis, compared with the scramble RNA negative control (NC). Bars represent the mean ± SD from three independent experiments (*n* = 3), each with three technical replicates. (**E**) Representative flow cytometry images. (**F**) Tumors grew in nude mice removed at day 28 after xenograft. (**G**) Knockdown of *Kidins220* inhibited tumor growth of xenograpted PC3 cells based on two-dimensional caliper measurements of the tumor size (upper panel) and the weight of those removed tumours (lower panel). Data represent mean ± SD, each group with five tumours (*n* = 5). Two independent experiments were performed and similar results were obtained. (**H**) *Kidins220* knockdown significantly decreased (*P* = 2.08×10^−2^) Ki-67 expression detected by immunostaining in tumours from xonografted PC3 cells with representative immunostaining images (original magnification, ×100) on the left. * indicates *P* < 0.05, and ** indicates *P* < 0.01 by Student's *t*-test, respectively.

To explore whether miR-4638-5p may suppress CRPC development by inhibiting Kidins220 expression, we firstly examined the expression of Kidins220 in the 30 ADPC and 18 CRPC samples as well as PCa cell lines using qRT-PCR, we found that Kidins220 expression was significantly much higher in CRPC tissues compared with ADPC tissues (*P* = 3.10 × 10^−7^; Figure 4G), which was reversely correlated with miR-4638-5p in those two groups of clinical samples. In addition, the mRNA of Kidins220 was also detected at higher levels in the androgen independent LNCaP C4-2B, PC3 and DU145 cells than in the androgen sensitive LNCaP cells (*P* < 0.05; Figure 4H). To further determine the cellular effect of Kidins220 in CRPC cells, we knocked down Kidins220 in PC3 and DU145 cells. We tested three Kidins220 shRNAs, which all showed knockdown effect at the protein levels, and selected shRNA (S-2), which achieved the best knockdown effect (Figure 5A) to establish stable Kidins220 knockdown cells (Figure 5B). As expected, compared with the pCDH-vector control cells, Kidins220-knockdown PC3 and DU145 cells showed a reduction in cell proliferation and the increase of apoptotic cells *in vitro* (*P* < 0.05; Figures 5C–5E) and reduced tumor growth in xenographed nude mice (*P* < 0.05; Figure 5F–5H), mimicking the effects of miR-4638-5p overexpression.

### Kidins220, mediating the effect of miR-4638-5p downregulation, promotes PCa neo-angiogenesis

**Figure 6:**
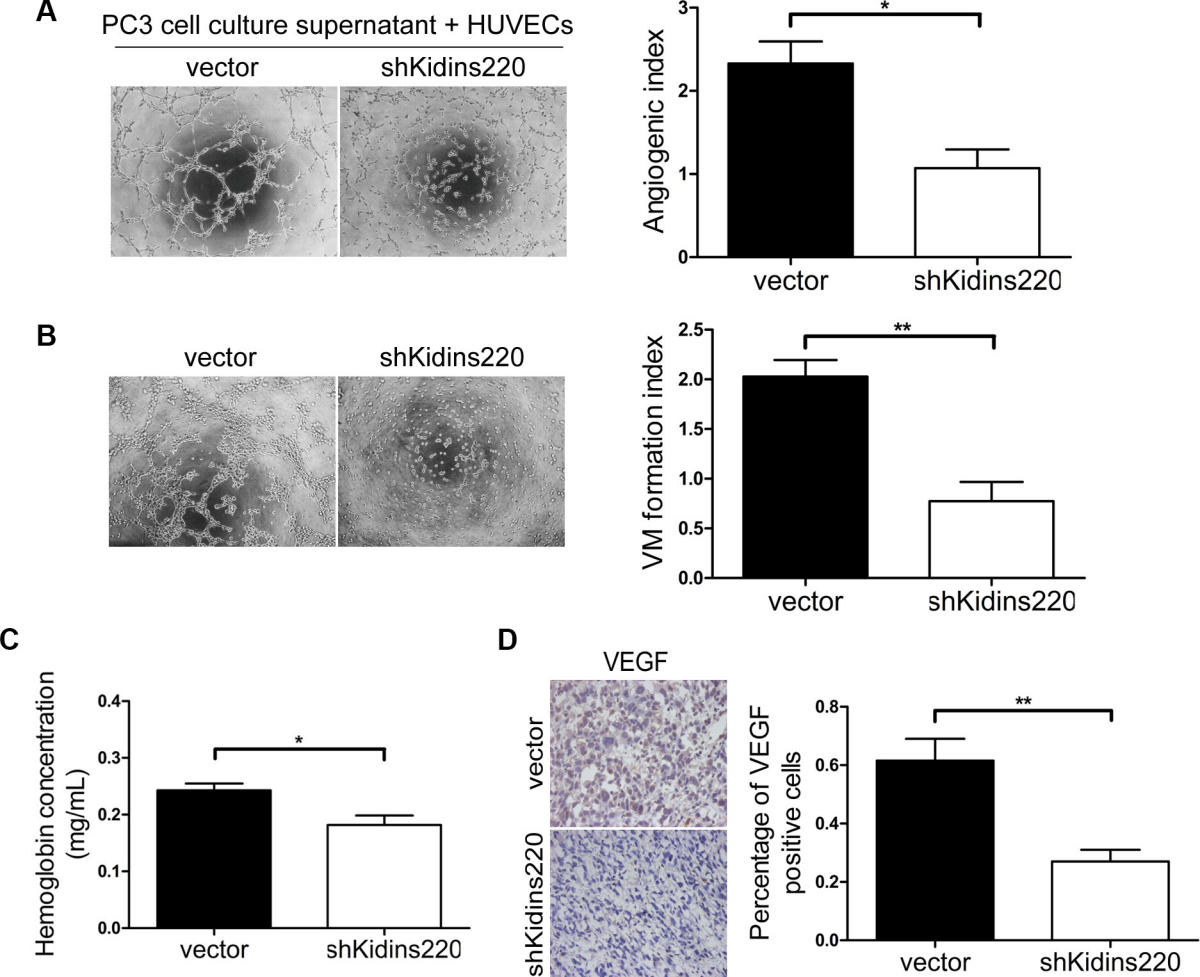
*Kidins220* knockdown inhibits the microtubule and vasculogenic mimicry formation of PCa cells and angiogenesis *in vivo* (**A**) Matrigel assay of microtubule formation. Tube formation assay was performed with HUVECs incubated in culture supernatant fluid of PC3 cells 48 hours after *Kidins220* knockdown or not. The photographs of microtubules were captured at 6 hour post seeding (original magnification, ×100). The quantification data represent the mean ± SD from three independent experiments (*n* = 3), each experiment containing four technical replicates. (**B**) Vasculogenic mimicry formation assay was performed with PC3 cells. The mimicry formation ability of PC3 cells transduced with the pCDH-sh*Kidins220* (shKidins220) was significantly decreased, compared to the cells transduced with the pCDH-vector controls (vector). Representative images of mimicry were captured at 6 hour post seeding (original magnification, ×100) on the left. The data represent the mean ± SD from three independent experiments (*n* = 3), each experiment containing four technical replicates. (**C**) The hemoglobin level of the Matrigel plugs in the PC3 xonografted tumours with Kidins220 knockdown was significantly decreased. Data represent mean ± SD, each group with five tumours (*n* = 5). Three independent experiments were performed with similar results. (**D**) *Kidins220* knockdown significantly decreased VEGF expression as detected by immunostaining in tumours from xonografted PC3 cells (*P* = 6.06 × 10^−3^) with representative immunostaining images (original magnification, ×100) on the left. * indicate *P* < 0.05 and ** indicate *P* < 0.01 by Student's *t*-test, respectively.

As Kidins220 have been reported to activate VEGFR [37], which play a key role in cancer neo-angiogenesis, we also investigated the role of Kidins220 in PCa angiogenesis. Microtubule formation assay was performed with HUVECs and conditioned medium collected from culture supernatant fluid of PC3 cells 48 hours after *Kidins220* knockdown by shRNA. We found that knockdown of *Kidins220* in PC3 cells decreased microtubule formation ability of HUVECs *in vitro* (*P* = 1.14 × 10^−2^; Figure 6A). Vasculogenic mimicry analysis was also performed with PC3 cells with and without *Kidins220* knockdown and we found that knockdown of *Kidins220* reduced 62% mimicry formation of PC3 cells (*P* = 2.83 × 10^−3^; Figure 6B). We also determined whether knockdown of *Kidins220* in the androgen-independent PC3 cells would inhibit angiogenesis *in vivo* by measuring the final hemoglobin content using Drabkin's reagent kit in the xenografts and found that hemoglobin content in the tumor xenografts of cells with *Kidins220* knockdown was significantly (*P* = 1.96 × 10^−2^) decreased compared with control cells transfected with non-targetting shRNA (Figure 6C). As expected, we detected significantly decreased VEGF expression in xenografts of *Kidins220* knockdown PC3 cells by immunostaining (*P* = 6.06 × 10^−3^; Figure 6D).

**Figure 7:**
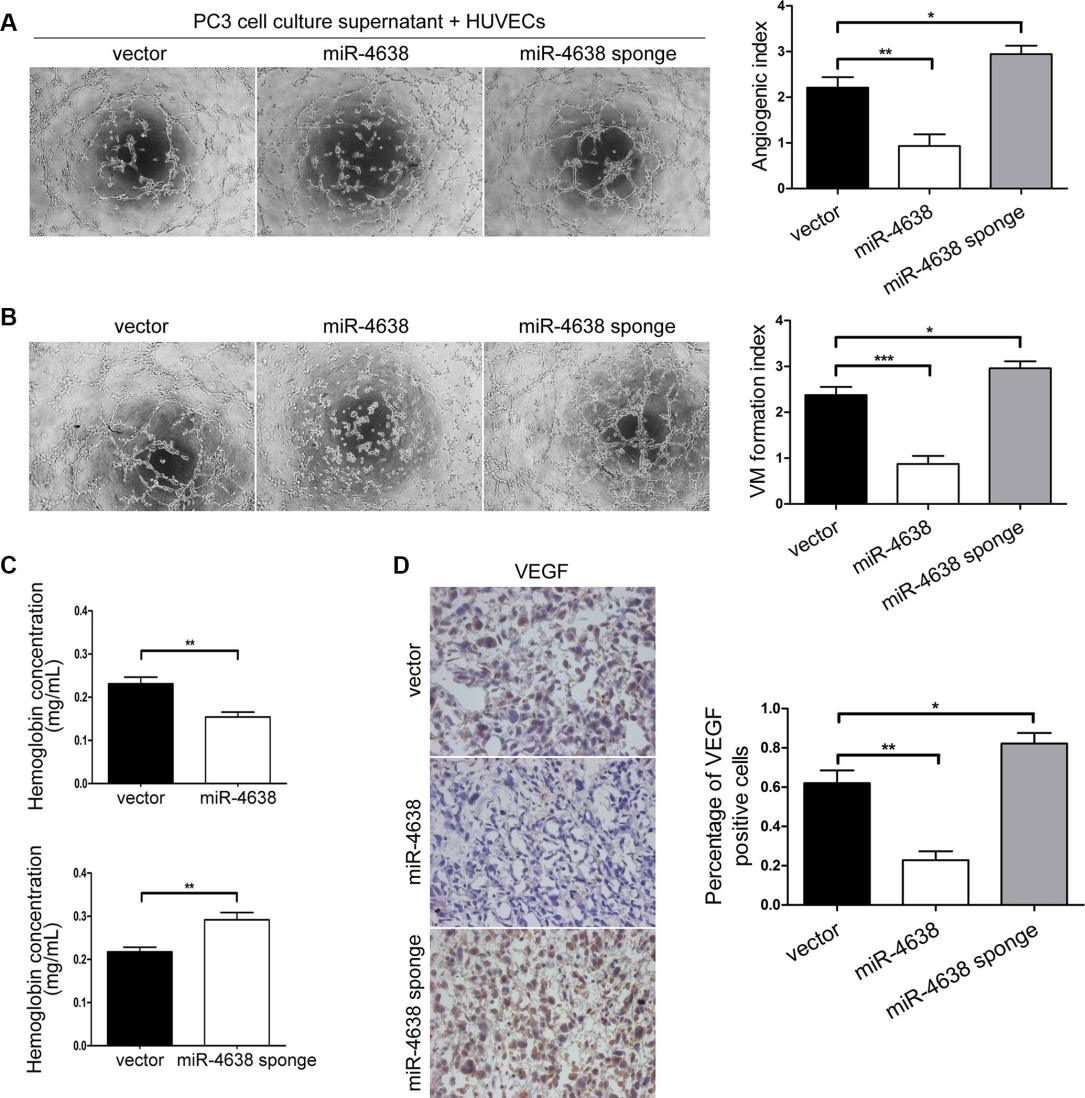
miR-4638-5p inhibits angiogenesis *in vitro* **and** *in vivo* **and mimicry formation of PCa cells.** (**A**) Matrigel assay of microtubule formation. Tube formation assay was performed with HUVECs cells incubated in culture supernatant fluid of PC3 cells 48 hours after transfection with the pCDH-miR-4638-5p (miR-4638) or the pCDH-miR-4638-5p sponge (miR-4638 sponge). The photographs of microtubules were captured at 6 hour post seeding (original magnification, ×100). The quantification data represent the mean ± SD from three independent experiments (*n* = 3), each experiment containing four technical replicates. (**B**) Representative mimicry formation images (original magnification, ×100) and bar chart showing that vasculogenic mimicry formation ability of PC3 cells transduced with the pCDH-miR-4638- 5p (miR-4638) or the pCDH-miR-4638-5p sponge (miR-4638 sponge) were significantly decreased or increased, respectively compared to the cells transduced with the pCDH-vector controls (vector). The data represent the mean ± SD from three independent experiments (*n* = 3), each experiment containing four technical replicates. (**C**) The hemoglobin level of the Matrigel plugs in the PC3 xonografted tumours with miR-4638-5p overexpression and inhibition was significantly decreased (*P* = 4.39 × 10^−3^) and increased (*P* = 7.87 × 10^−3^), respectively. Data represent mean ± SD, each group with five tumours (*n* = 5). Three independent experiments were performed with similar results. (**D**) miR-4638-5p overexpression and inhibition also significantly decreased (*P* = 1.60 × 10^−3^) and increased (*P* = 4.51 × 10^− 2^), respectively, VEGF expression detected by immunostaining in tumours from xonografted PC3 cells. Representative immunostaining images (original magnification, ×100) on the left. *, ** and *** indicate *P* < 0.05, *P* < 0.01 and *P* < 0.001 by Student's *t*-test, respectively.

Following the finding that Kidins220 influences PCa angiogenesis, we explored whether miR-4638- 5p suppresses PCa angiogenesis both by inhibiting microtubule formation of endothelial cells through VEGF paracrine secretion and the formation of vasculogenic mimicry by PCa cells. The microtubule formation ability of HUVECs incubated with culture supernatant of PC3 cells overexpressing miR-4638-5p was significantly (*P* = 9.92 × 10^−3^) weaker than cells incubated with culture supernatant of those PCa cells transduced with the pCDH-vector control. HUVECs incubated with culture supernatant of PC3 cells transduced with pCDH-miR-4638-5p sponge showed significantly (*P* = 4.74 × 10^−2^) higher angiogenesis ability compared with the pCDH-vector control (Figures 7A), indicating that miR-4638-5p may block the microtubule formation ability of HUVECs through VEGF paracrine secretion in PC3 cells. We also performed vasculogenic mimicry analysis of PC3 cells with different level of miR-4638-5p. Vasculogenic mimicry formation ability of PC3 cells transduced with the pCDH-miR-4638-5p or pCDH-miR-4638-5p sponge were significantly decreased (*P* = 9.42 × 10^−4^) or increased (*P* = 4.79 × 10^−2^), compared to the cells transduced with the pCDH-vector control (Figures 7B). Furthermore, our *in vivo* experiments using tumor xenografts from PC3 cells showed that the final hemoglobin content in tumor xenografts of PC3 cells transduced with the pCDH-miR-4638-5p was significantly (*P* = 4.39 × 10^−3^) decreased compared with the empty vector transduced control cells (Figure 7C). The opposite effect was observed with cells transduced with pCDH-miR-4638-5p sponge in comparison to the control cells (*P* = 7.87 × 10^−3^) (Figure 7C). MiR-4638-5p overexpression and inhibition also significantly decreased (*P* = 1.60 × 10^−3^) and increased (*P* = 4.51 × 10^−2^), respectively, VEGF expression in xonografted PC3 cells as detected by immunostaining (Figure 7D). Therefore, the phenotype observed upon miR-4638-5p overexpression and *Kidins220* knockdown were similar, suggesting that miR-4638-5p may target *Kidins220* to regulate not only androgen independent tumor growth but also PCa angiogenesis both through stimulating endothelial cells and forming vasculogenic mimicry.

### PI3K/AKT pathway may mediate KIDINS220-induced androgen independent PCa growth and angiogenesis

**Figure 8:**
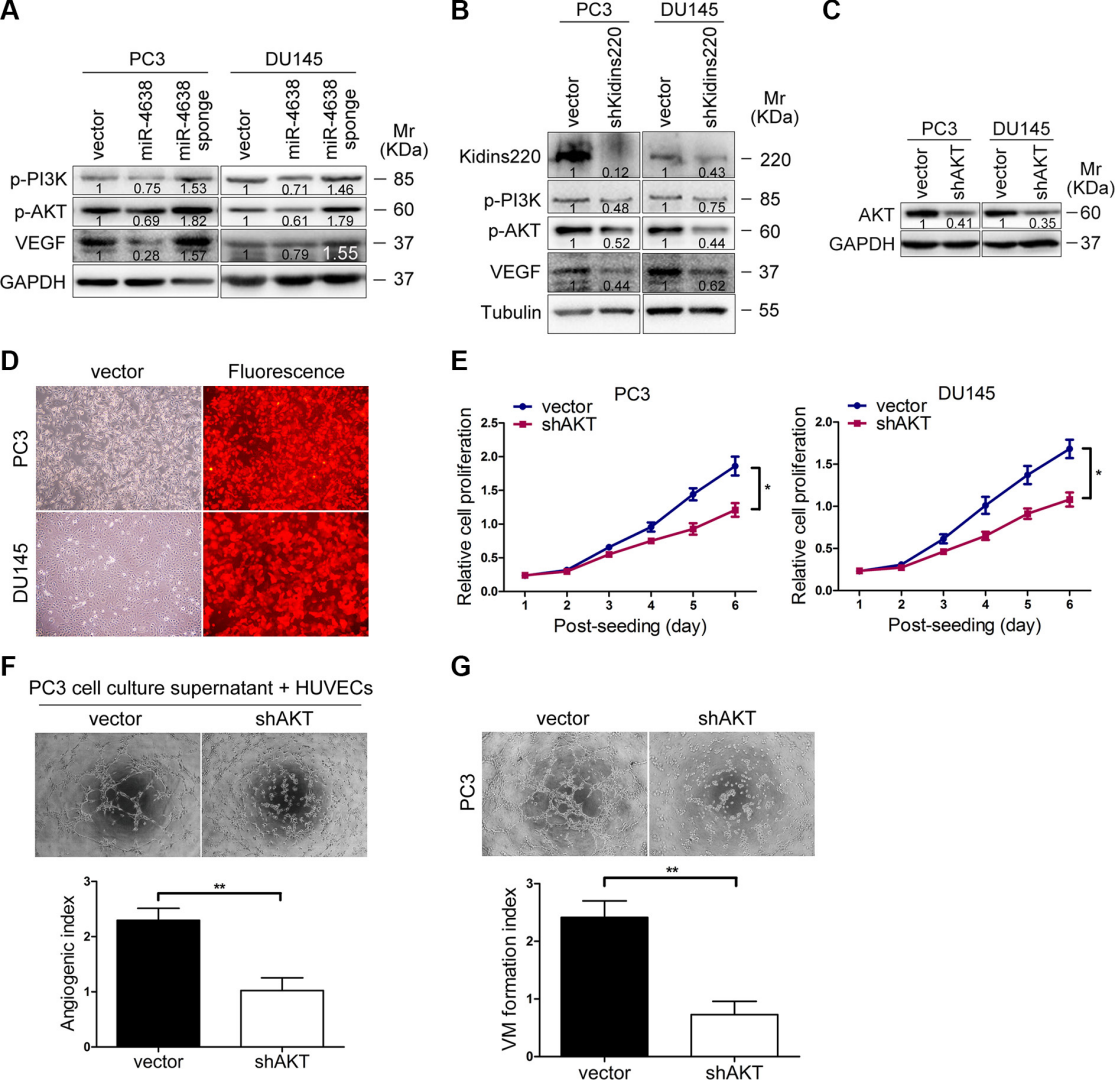
PI3K/AKT signaling pathway mediates Kidins220-induced tumor growth, angiogenesis and mimicry formation of PCa cells (**A**) Western blot analysis of VEGF and phosphorylation levels of PI3K, AKT (p-PI3K and p-AKT, respectively) in PC3 and DU145 cells transduced with pCDH-miR-4638-5p (miR-4638) or pCDH-miR-4638-5p sponge (miR-4638 sponge). Numbers labeled under the bands were the relative intensities of the bands after calibrating for loading with the house-keeping protein. The relative value of proteins in the vector group was considered as ‘1′. (**B**) Western blot analysis of total Kidins220, VEGF and phosphorylation levels of PI3K and AKT (p-PI3K and p-AKT, respectively) in PC3 and DU145 cells transduced with the pCDH-sh*Kidins220* and the controls. Numbers labeled under the bands were the relative intensities of the bands after calibrating for loading with the house-keeping protein. The relative value of proteins in the vector group was considered as ‘1′. (**C**) Western blot analysis of total AKT levels in PC3 and DU145 cells transduced with the pCDH-sh*AKT* or not. Numbers labeled under the bands were the relative intensities of the bands after calibrating for loading with the house-keeping protein. The relative value of proteins in the vector group was considered as ‘1′. (**D**) The expression of RFP observed under the fluorescence microscope 72 hours after PC3 and DU145 cells were infected with the pCDH-sh*AKT* and the pCDH-vector control under the same multiplicity of infection. (original magnification, ×100). (**E**) Cell proliferation results of PC3 and DU145 cells transduced with the pCDH-sh*AKT* (shAKT) or the pCDH-vector controls (vector), showing the reduced proliferative capacity of both PC3 and DU145 cells after stably underexpressing *AKT*. Data represent mean ± SD determined from three independent experiments (*n* = 3), each with four technical replicates. (**F**) Matrigel analysis of microtubule formation. Tube formation assay was performed with HUVECs cells incubated in culture supernatant fluid of PC3 cells 48 hours after *AKT* knockdown or not. The photographs of microtubules were captured at 6 hour post seeding (original magnification, ×100) on the top. Quantification of results was performed subsequently. The data represent the mean ± SD from three independent experiments (*n* = 3), each experiment containing four technical replicates. (**G**) Vasculogenic mimicry formation assay was performed with PC3 cells. The mimicry formation ability of PC3 cells transduced with the pCDH-sh*AKT* (shAKT) was significantly decreased, compared to the cells transduced with the pCDH-vector controls (vector) with representative images of mimicry captured at 6 hour post seeding (original magnification, ×100) on the top. The data represent the mean ± SD from three independent experiments (*n* = 3), each experiment containing four technical replicates. * indicate *P* < 0.05 and ** indicate *P* < 0.01 by Student's *t*-test, respectively.

**Figure 9:**
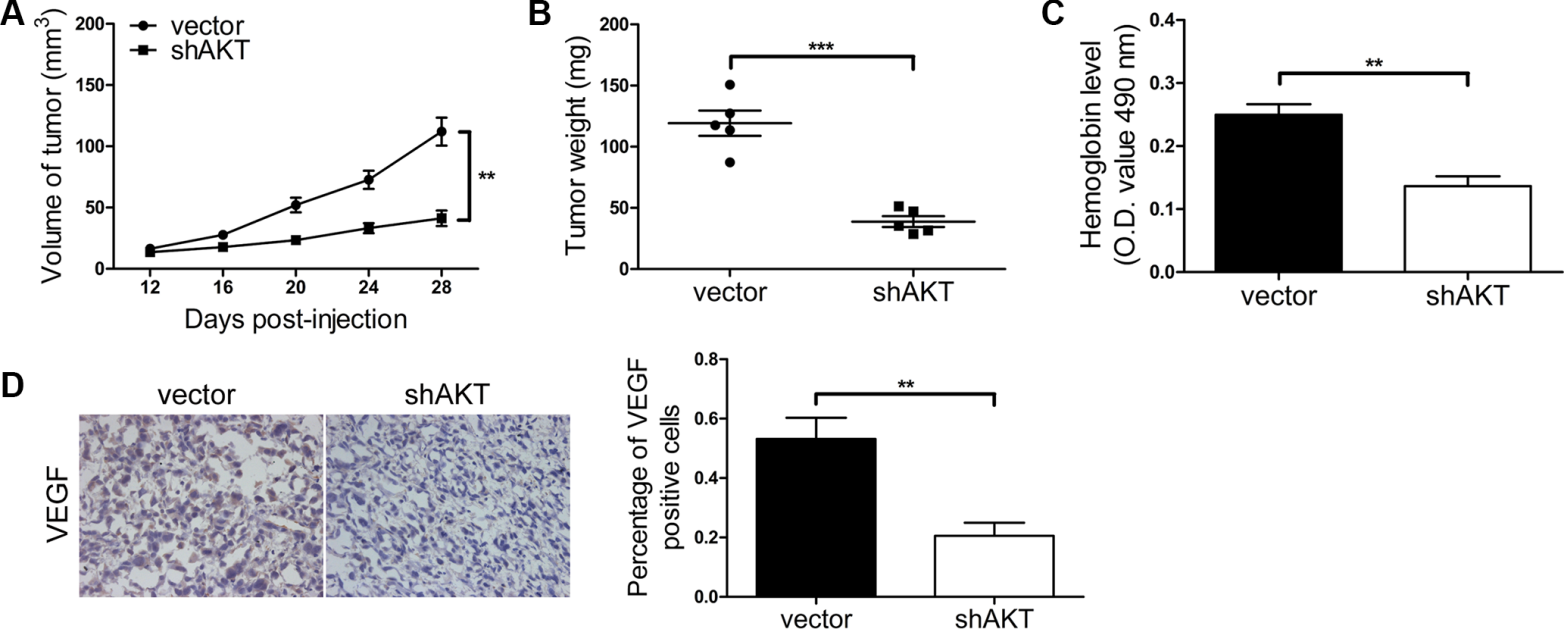
PI3K/AKT signaling pathway mediates Kidins220-induced tumorigenesis and angiogenesis *in vivo.* (**A–B**) *AKT* knockdown reduced tumorigenesis *in vivo*. PC3 cells were transduced with the pCDH-sh*AKT* (shAKT) or the pCDH-vector control (vector) for 72 hour and further re-suspended in serum-free medium. The sizes of tumors from nude mice were determined by two-dimensional caliper measurements. Scatter plots represent the weights of independent tumors from different groups. Data represent mean ± SD, each group with five tumors (*n* = 5). Two independent experiments were performed and similar results were obtained. (**C**) The hemoglobin level of the Matrigel plugs was determined with O.D. value at 540 nm. Data represent mean ± SD and each group with five tumors (*n* = 5). Three independent experiments were performed and similar results were obtained. (**D**) *AKT* knockdown significantly decreased VEGF expression detected by immunostaining in tumors from xonografted PC3 cells (*P* = 6.29 × 10^−3^) with representative immunostaining images (original magnification, ×100) shown on the left. ** and *** indicate *P* < 0.01 and *P* < 0.001 by Student's *t*-test, respectively.

To further investigate cellular signaling pathway regulated by miR-4638-5p and sh*Kidins220*, we performed Western blotting for candidate downstream genes responsible for CRPC and angiogenesis, in the PC3 and DU145 cells transfected with the pCDH-miR-4638-5p, pCDH-miR-4638-5p sponge or empty pCDH-vector control. We found that stable transduction with the pCDH-miR-4638-5p or pCDH-miR-4638-5p sponge resulted in the reduction or increase, respectively, of VEGF as well as phosphorylated forms of PI3K and AKT (p-PI3K and p-AKT, respectively) (Figure 8A). Consistent with the results of miR-4638-5p manipulating, *Kidins220* knockdown by lentivirus mediated sh*Kidins220* transfection similarly inhibited the VEGF, p-PI3K and p-AKT protein expression (Figure 8B). To further investigate whether PI3K/AKT pathway mediates Kidins220-induced tumor growth and angiogenesis, we knocked down *AKT* with shRNA (Figure 8C and 8D). As expected, compared with the pCDH-vector transfected control cells, *AKT*-knockdown PC3 and DU145 cells showed reduction in cancer cell growth (*P* < 0.05 for all; Figure 8E and Figure 9A–9B) and angiogenesis (*P* < 0.01 for all; Figure 8F and Figure 9C–9D) *in vitro* and *in vivo*, similar to the phenotype observed upon *Kidins220*-knockdown. Meanwhile, vasculogenic mimicry formation ability of PC3 cells transduced with sh*AKT* was significantly decreased (*P* = 4.22 × 10^−3^), compared to the cells transduced with the pCDH-vector control (Figure 8G). Taken together, these results suggest that PI3K/AKT pathway may mediate Kidins220-induced angiogenesis and vasculogenic mimicry in addition to PCa cell proliferation and castration resistance.

## DISCUSSION

CRPC is the main cause of mortality from PCa. Our current knowledge on CRPC development is mainly focused on androgen metabolism and androgen receptor pathways [38]. Identification of other molecular changes associated with CRPC will greatly help us to control this common and lethal form of male malignancy. Alterations of many miRNAs have been reported in PCa development, progression as well as response to therapies [39]. The role of miRNAs in androgen signaling and CRPC development has also been investigated in cell lines and animal models [40]. Differential expression of miRNAs between CRPC and ADPC samples have also been reported [41–44]. Here, using miRNA microarray analysis, we identified a number of differentially expressed miRNAs between Chinese ADPCs and CRPCs. While many of them have been previously studied, miR-4638-5p has not been investigated previously in any human cancers. The investigation of the down-regulation of miR-4638- 5p in PCa was also supported by its down-regulation in advanced disease [33] and PCa stem cells from separate miRNA microarray data. MiR-4638-5p was the mostly underexpressed miRNA in isolated stem cells compared to non-stem DU145 cells. PCa stem cell are closely associated with castration resistance, that PCa stem cell markers have been associated with the development of CRPC, such as CD166 [45], Nanog [46, 47], Bmi-1 [48] and Sox2 [49, 50]. We also further confirmed the downregulation of miR-4638-5p in CRPC samples using a larger series of clinical samples and demonstrated both through *in vitro* and *in vivo* studies that miR-4638-5p play a significant role in suppressing androgen independent PCa cell growth.

The function of miR-4638-5p is currently unknown; the only published report on miR-4638-5p was from a bioinformatics analysis of potential intronic and 3′UTR targeted miRNAs from known cardiac marker genes, where miR-4638-5p has been supposed to regulate cardiac marker gene *MYH-7*, which encodes beta-myosin heavy chain and is expressed in cardiac and skeletal muscle tissues [51]. Here we demonstrated that miR-4638-5p is a putative tumor suppressor, which suppresses PCa growth, angiogenesis and castration resistance development through regulating Kidins220, VEGFR and PI3K/AKT pathway. As miRNA has been developed for therapeutic use [52], miR-4638-5p may represent a novel target for CRPC treatment. The miR-4638-5p level change, as a driver, may occurs before the clinical features of CRPC, thus it has the potential to be developed into a biomarker for the onset of CRPC before PSA increase or other clinical progressions.

In the search for genes/proteins regulated by miR-4638-5p, we identified *Kidins220* as a gene whose expression was downregulated post-transcriptionally by miR-4638-5p through targeting the *Kidins220* 3′- UTR seed sequences. In consistent to miR-4638-5p under-expression, we demonstrated that CRPC samples overexpressed *Kidins220* compared to ADPC samples and knockdown of *Kidins220* generated similar effects as miR- 4638-5p overexpression in CRPC cells.

Kidins220 (kinase D–interacting substrate of 220 kDa), also known as ankyrin repeat-rich membrane spanning protein (ARMS), is a recently identified tetra-spanning-transmembrane protein abundantly expressed in the developing and adult neural tissues [53, 54]. Previous studies of Kidins220 were mainly focused on its roles in neural development and Kidins220, as a downstream substrate of protein kinase, neurotrophin and ephrin, regulates neuronal differentiation and survival [55–58]. Kidins220 also expresses outside the nervous system. It interacts with the microtubule and actin cytoskeleton, modulating cell plasticity and migration and is important for cardiovascular development and certain immune function [58–60]. Although most of the above cellular features are associated with cancer development and progression, research of Kidins220 in human malignancies is very limited. *Kidins220* overexpression has been reported in melanoma and it has been shown to contribute to tumor formation by activating MEK/ERK signaling pathway and consequently preventing transformed melanocytes from the stress-induced apoptosis [61]. Expression of Kidins220 has also been reported in neuroblastoma tumours, where it stabilizes nerve growth factor-induced survival signalling through MAPK/ERK signalling [62]. Both of these studies demonstrated that Kidins220 protects cells for survival through the MAPK/ERK signalling pathway. However, the role of Kidins220 in PCa has not been reported. In this study we demonstrated that Kidins220 promoted CRPC cell growth and tumor angiogenesis and enhanced VEGFR, PI3K and AKT activity in CRPC cancer cells. These findings suggest that, in addition to the above reported roles, Kidins220 impacts on PI3K/AKT and VEGF/VEGFR pathways to promote PCa angiogenesis and the development of castration resistance. MiR- 4638- 5p, to our knowledge, is also the first identified miRNA to regulate *Kidins220*. Overall, these novel findings increase our understanding of the cellular and biological functions of miR-4638-5p and Kidins220 as well as reveal new mechanisms underlying the development of CRPC.

The PI3K/AKT signaling is a critical pathway in cell proliferation, survival, neovascularization and tumor growth [63, 64]. Previous studies have identified that PI3K/AKT signaling plays a key role in the development and maintenance of CRPC [65, 66]. Deregulation of PI3K/AKT signaling occurs in almost all cases of advanced CRPC [67]. Recent studies have revealed a direct link between PI3K/AKT and AR signaling pathways that they interplay during the development of castration resistance [68, 69]. In addition, the activation of PI3K/AKT signaling pathway promotes castration resistance and the inhibition of AR function accelerates the development of CRPC in PTEN deficient mice [70]. As mentioned above, the known function of Kidins220 in cancer is to protect cells for survival when they are under stress [61, 62]. In PCa, Kidins220 may activate PI3K/AKT signaling to sustain cancer cell growth under the stress of androgen deprivation and lead to CRPC. Inhibiting a single target in the PI3K/AKT signaling pathway have shown limited clinical efficacy and simultaneously inhibition of multiple targets in series or parallel have demonstrated greater promise in the treatment of CRPC [65]. As Kidins220 regulates both PI3K and AKT activities, targeting miRNA-4638- 5p and/or Kidins220 offers another promising therapeutic approach for the management of CRPC.

**Figure 10:**
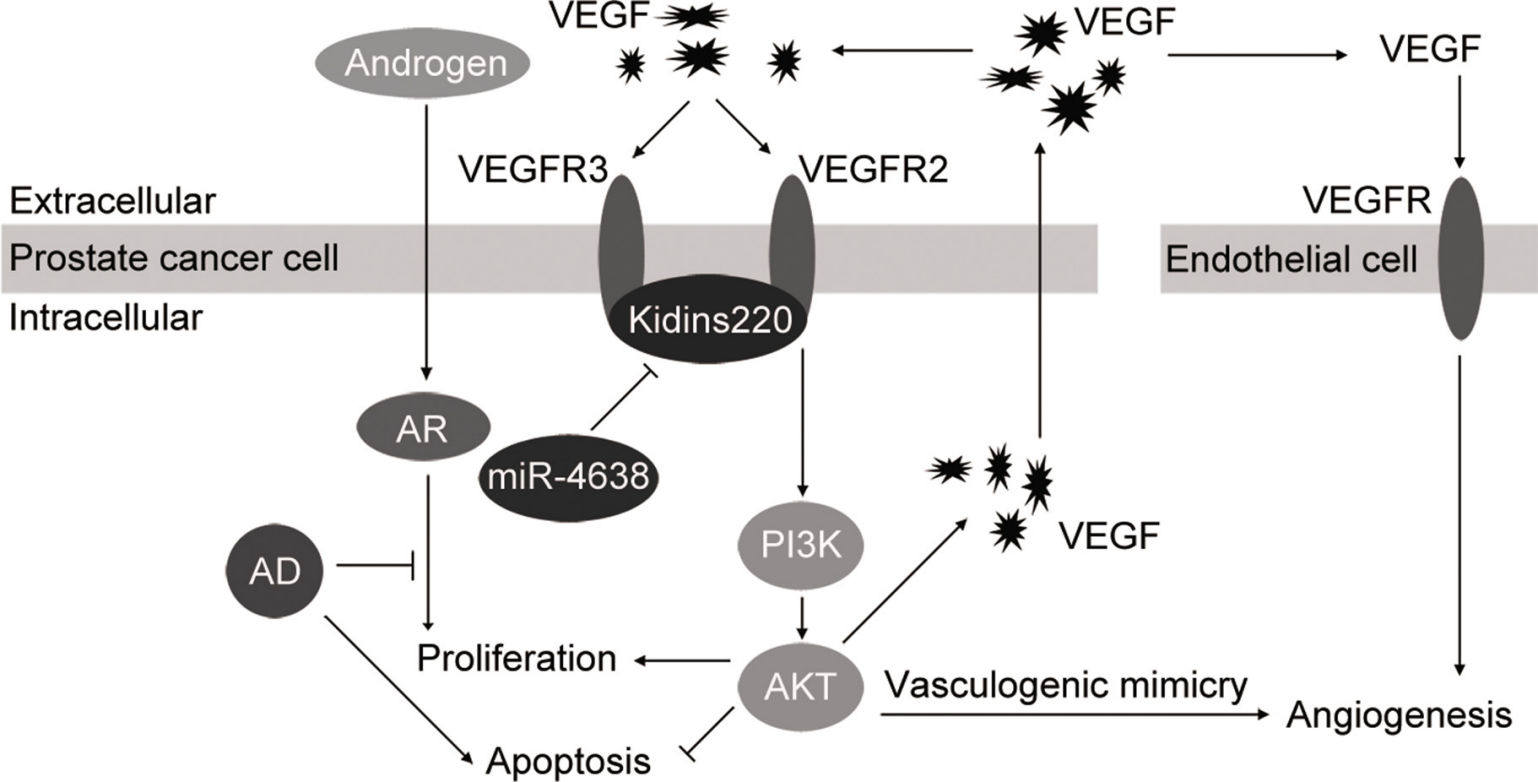
Proposed model of miR-4638-5p in control cell growth and angiogenesis in prostate cancer cell Kidins220, regulated by miR-4638-5p, binds to the VEGFR2 and VEGFR3 and promotes interaction between VEGF and VEGFR as a co-receptor (auxiliary receptors) of VEGFR, so that VEGF/PI3K/AKT signaling pathway is activated to promote proliferation and mimicry formation and suppress apoptosis of PCa cells. AKT activation also consequently increases the expression level and secretion of VEGF, which stimulates angiogenesis of endothelial cells. Therefore, miR-4638-5p inhibits these cellular process by downregulating its target gene *Kidins220*.

In this study, we provided evidence that Kidins220 modulated angiogenesis of PCa cells via regulation of both the VEGF/VEGFR2 and PI3K/AKT signaling pathway. It has been reported that Kidins220 interacts with VEGFR2 and VEGFR3 in mouse endothelial cells and modulates VEGF signaling through promoting the interaction between VEGF and VEGFR for cardiovascular development [37]. VEGF is the main angiogenic growth factor [71] and PI3K/AKT signaling interplay with VEGF level to regulate angiogenesis [64], which is critical for tumor development when cancer reaches to a certain size. Numerous studies have demonstrated that Hif-1α activation could increase the expression and secretion of VEGF via PI3K/AKT/mTOR signaling pathway [72–74]. Meanwhile, VEGF itself can effectively activate PI3K/AKT signaling pathway, stimulating blood vessel in autocrine and paracrine manner. Therefore, the observation that Kidins220 regulated angiogenesis in human cancers via activation of VEGF and PI3K/AKT signaling is not surprising, but this has not been investigated before. In this study, we also showed for the first time that Kidins220 through VEGF and PI3K/AKT signaling regulates both neo-vascularization from endothelial cells and the formation of vasculogenic mimicry of CRPC cells for tumor angiogenesis. Vasculogenic mimicry (VM) phenomenon provides a new pathway for tumor blood supply independent of endothelial cells [75]. The tumor cells possessing mimic vessel display highly malignant characteristics of invasive growth, dedifferentiation and metastasis [76]. The ability of vasculogenic mimicry formation of cancer cells may be an additional mechanism for them to access sufficient nutrition and growth factors for cell survival and proliferation under adverse stressful environment, such as androgen deprivation in the case of CRPC. Based on our results, a model of miR-4638-5p and Kidins220 functions for cell survival and angiogenesis has been proposed (Figure 10).

While angiogenesis is critical for cancer progression and anti-angiogenesis therapy has been extensively tested with certain success in treating CRPC [77], the role of angiogenesis in CRPC development has not been reported. Therefore, the role of accelerated angiogenesis by miRNA-4638-5p down-regulation and the consequently deregulation of the above molecular pathways in castration development is unclear. Angiogenesis may provide oxygen, nutrition and other growth factors to help the survival of cancer cells under stress of androgen depletion during CRPC development. Neuroendocrine PCa is AR negative and with poor prognosis, therefore, the development of neuroendocrine PCa has been considered as a mechanism of CRPC [78]. Kidins220 is critical for neuronal differentiation and survival [37, 55, 57–59]. As we have demonstrated *in vitro* and *in vivo* that Kidins220 promote tumor angiogenesis through activation of PI3K/AKT and VEGF signalling, Kiddins220 may also involved in both neuroendocrine differentiation and angiogenesis of PCa cells. Both neuroendocrine differentiation and vasculogenic mimicry formation are cellular changes of losing epithelial features of PCa cells. Kiddins220 may coordinate neuroendocrine differentiation and angiogenesis, together with regulating PI3K/AKT signaling, promoting the development of castration resistance. Further investigations are required.

In summary, we identified a novel tumor suppressor miRNA, miR-4638-5p, which is downregulated in CRPC, and dissected its target genes and downstream cell signaling pathways. We revealed for the first time that miR-4638-5p regulates *Kidins220* mRNA by targeted degradation and consequently suppression of VEGF/VEGFR and PI3K/AKT in CRPC cells, and impact on tumor growth and angiogenesis using *in vitro* and *in vivo* models. While the role of PI3K/AKT pathway in CRPC initiation is well established, the role of VEGF signaling and angiogenesis in CRPC development warrants further investigations. These findings provide new molecular insights into the development of CRPC and can guide the development of novel approaches targeting the miR- 4638- 5p/Kidins220/VEGF/VEGFR/PI3K/AKT axis to prevent or treat CRPC.

## MATERIAL AND METHODS

### Human prostate tissue specimens

Fresh frozen tissue from 30 ADPC and 18 CRPC patients were obtained from Wuxi No. 2 People's Hospital affiliated with Nanjing Medical University. The 18 CRPC patients were selected based on their increased serum prostate-specific antigen (PSA) level during androgen-deprivation therapy and the cancer tissues were obtained through transurethral prostatic resection to relieve the symptom of urinary obstruction. The 30 ADPC samples were derived from radical prostatectomy in treatment-naive patients. Clinical stage of disease was determined by histopathology, magnetic resonance image and radio-nucleotide bone scans according to the 2002 TNM classification of prostate carcinoma. The clinic-pathological features of all study patients are summarized in Supplementary Table S1. All these samples contained more than 60% tumor by pathological examination. The use of these human PCa tissues for this study was approved by the institutional ethics review board of Nanjing Medical University.

### Cell lines

Human umbilical vein endothelial cells (HUVECs) were maintained in EBM-2 culture media (LONZA, Allendale, NJ, USA) as previously described [79]. DU145 human androgen independent PCa cell line and HEK293T were maintained in DMEM, and androgen dependent LNCaP, androgen independent LNCaP C4-2B subline and PC3 human PCa cell lines was cultured in RPMI 1640, all supplemented with 10% fetal bovine serum (FBS), 100 units/ml of penicillin and 100 μg/ml of streptomycin.

### MiRNA microarray analysis

Total RNA was isolated using TRizol (Invitrogen) and purified with RNeasy mini kit (QIAGEN) according to manufacturer's instructions. RNA quality and quantity was measured using NanoDrop spectrophotometer (ND-1000, NanoDrop Technologies) and RNA Integrity was determined by gel electrophoresis. 2.5 μg total RNA samples were labeled using the miRCURYTM Array Power Labeling kit (Exiqon, Vedbaek, Denmark) following the manufacturer's instruction. Microarray images were acquired using a Genepix 4000B scanner (Axon Instruments, Union City, CA, USA), processed and analyzed with Genepix Pro 6.0 software (Axon Instruments).

### Plasmids and miRNA mimics

The miR-4638-5p (Invitrogen, Shanghai, China), *Kidings220* shRNA (Invitrogen) and *AKT* shRNA, (Invitrogen), were cloned into the lentiviral vector *pCDH-CMV-MCS-EF1-tRFP* with modification as previously described [80] and a miR-4638-5p sponge (Invitrogen) as described below was cloned into another lentiviral vector *pCDH-CMV-MCS-EF1-copGFP* (System Bioscience, CA, USA) for stable miR-4638-5p, *Kidings220* and *AKT* overexpression and/or knockdown studies together with the empty lentiviral vector (pCDH-vector control). Synthetic miR-4638-5p mimic (miR-4638 mimic, RIBOBIO, Guangzhou, China), miR-4638-5p inhibitor (miR-4638 inhibitor, RIBOBIO), *Kidings220* siRNA (si*Kidins220*, RIBOBIO) and corresponding scramble RNA negative controls (RIBOBIO) were applied for transient overexpression or knockdown analyses. pGL3 luciferase reporter control plasmid (pGL3-Control, Promega, Shanghai, China), pGL3-Luc-*Kidins220-*3′-UTR WT and pGL3-Luc-*Kidins220*-3′-UTR Mut reporter plasmids as described below, miR-4638 mimic and miR-4638 mutation mimic (RIBOBIO) were used for luciferase reporter assay to investigate *Kidins220* promoter activity.

To generate miR-4638-5p lentiviral constructs (pCDH-miR-4638-5p), the sequence of miR-4638-5p (5′-ACTCGGCTGCGGTGGACAAGT-3′), obtained from the miRBase, and its complementary sequence with two mutation sites (5′-TCTTGTCCACCGGAGCCGAGT-3′) were inserted between the stem-loop structure and the flanking sequence of pre-miR-30 as previously described [80], to obtain the precursor stem-loops of miR-4638-5p, which was synthesized by Invitrogen and used as the PCR template sequence to be amplified by the following PCR primers designed using Primer Premier 5.0 software, forward: 5′-CAGAAGGCTCG AGAAGGTATATTGCTGTTGACAGTGAGCG-3′ (with Xho I sequence underlined) and Reverse: 5′-CTAAAG TGACCCCTTGAATTCCGAGGCAGTAGGCA-3′ (with EcoR I sequence underlined). The amplified precursor stem-loops of miR-4638-5p was cloned into the Xho I and EcoR I sites of the lentiviral vector *pCDH-CMV-MCS-EF1-tRFP*.

For the purpose of long-term stable downregulation of miR-4638-5p, miRNA sponge was produced by linking six complementary sequences of miR-4638-5p, germinated four consecutive restrictive mutations with five linker sequences to generate the miR-4638-5p sponge: 5′-ACTTGTCCAGGCGAGCCGAGTCGATACTTGTCC AGGCGAGCCGAGTACCGGTACTTGTCCAGGCGAG CCGAGTCGATACTTGTCCAGGCGAGCCGAGTCGA TACTTGTCCAGGCGAGCCGAGTACCGGTACTTGT CCAGGCGAGCCGAGT-3′, which was synthesized by Invitrogen. The miR-4638-5p sponge was cloned from the template plasmid *pUC57-AMP*^+^ into the EcoR I and BamH I sites of the lentiviral vector *pCDH-CMV-MCS-EF1-copGFP* to generate miR-4638-5p sponge lentiviral constructs (pCDH-miR-4638-5p sponge).

To further conform that the miR-4638-5p expression construct is functional in prostate cancer cells, the pGL3-miR-4638-5p sensor reporter (pGL3-miR-4638 Sensor for short) was produced by linking three tandem miR-4638-5p seed binding sequences and inserted into the downstream of luciferase sequence in the pGL3-Control plasmid. The sequence is as following: (forward) 5′-ACTTGTCC ACCGCAGCCGAGTAAGCTTACTTGTCCACCGCAG CCGAGTAAGCTTACTTGTCCACCGCAGCCGAGT-3′ and (reverse) 5′-ACTCGGCTGCGGTGGACAAGTAA GCTTACTCGGCTGCGGTGGACAAGTAAGCTTACT CGGCTGCGGTGGACAAGT-3′.

To stably knockdown *Kidins220,* the following three shRNAs for *Kidins220* were designed using bioinformatics software including siDirect, Integrated DNA Technologies, Learning with Clontech and SDS (siRNA Design Software).

shRNA-01: 5′-ATATGCAAGGGAGTATCTCCT-3′;

shRNA-02: 5′-AGTTAACCCCACATTTCAGTA-3′;

shRNA-03: 5′-ATTCTCATTCTGAATAGTGGT-3′. The sequence of shRNAs of *Kidins220* and their complementary sequences with one mutation site (5′-TGGA GATACTCCCTTGCATAT-3′, 5′-AACTGAAATGTGG GGTTAACT-3′, 5′-TCCACTATTCAGAATGAGAAT-3′) were inserted between the stem-loop structure and the flanking sequence of pre-miR-30 as previously described [80]. The shRNA sequences were amplified by PCR and cloned into the Xho I and EcoR I sites of the lentiviral vector *pCDH-CMV-MCS-EF1-tRFP* to produce pCDH-sh*Kidins220*. The same approach was used to make the AKT shRNA lentiviral construct, pCDH-sh*AKT*, to knockdown AKT.

### Cell transfections

For transient transfection of miR-4638 mimic, miR-4638 inhibitor, promoter constructs and siRNA of *Kidins220*, cells of 50%-60% confluence were transfected using Lipofectamine 2000 reagent (Invitrogen) according to the manufacturer's instruction.

To generate stable expression cell lines, the virus-packaging cells HEK293T (1 × 10^6^) were seeded in a 10 cm dish one day before co-transfection with pCDH-miR-4638-5p, pCDH-miR-4638-5p sponge, pCDH-sh*Kidins220* and pCDH-sh*AKT*, packaging plasmid *psPAX2* and envelope plasmid *pMD2.G*. 48 hours after transfection, the virus containing supernatant was collected and filtered using 0.45 μm filter to generate Lentivirus-pCDH-miR-4638-5p, Lentivirus-pCDH-miR-4638-5p sponge, Lentivirus-pCDH-sh*Kidins220* and Lentivirus-pCDH-sh*AKT*. The viral titer was determined by observing the expression of red or enhanced green fluorescent protein (RFP or EGFP). DU145 and PC3 cells were infected with these lentivirus under the same multiplicity of infection. The expression of RFP and EGFP was observed under the fluorescence microscope after 72 hours.

### Quantitative real-time PCR analysis of miRNA and mRNAs

Total RNA was extracted from PCa tissues and cultured cells using the TRizol method (Invitrogen) and subjected to the TaqMan® MicroRNA Reverse Transcription Kit (Applied Biosystems, Carlsbad, CA, USA) or the Promega Reverse Transcription Kit (Promega, Madison, WI) to obtain cDNA. TaqMan MicroRNA (Assay ID: 462195_mat for has-miR-4638- 5p) and gene expression (Assay ID: Hs01057000_m1 for *Kidins220*) assays (Applied Biosystems) were performed in accordance with the manufacturer's instructions using a 7500 Real-time PCR System (Applied Biosystems). The expression of U6 snRNA (Assay ID: 001973) was used as a control for miRNA expression and *GAPDH* (Assay ID: Hs02758991_g1) was used as a control for gene expression. The relative expressions were calculated using the 2^−ΔΔCt^ method.

### Cell proliferation assay

Cell Count Kit-8 (CCK-8) (Dojindo Molecular Technologies, Tokyo, Japan) was used to evaluate cell proliferation. PC3 and DU145 cells stably transfected with pCDH-miR-4638-5p, pCDH-miR-4638-5p sponge, pCDH*-*sh*Kidins220*, pCDH*-*sh*AKT* and pCDH-vector controls were seeded into 96-well plates at density of 2 × 10^3^ cells per well and cultured for 1 day, 2 days, 3 days, 4 days, 5 days and 6 days. Each well of cells were then cultured in 20 μl of CCK-8 solution in addition to the 200 μl of culture medium for 1.5 hours at 37°C. Absorbance at 450 nm was measured using a V_max_ microplate spectrophotometer (Molecular Devices). LNCaP cells transfected with pCDH-miR-4638-5p sponge and pCDH-vector control were seeded into 96-well plates (also 2 × 10^3^ cells per well) in the RPMI 1640 medium supplemented with 10% charcoal-stripped FBS and cultured for 1 day, 2 days, 3 days, 4 days and 5 days. Then the cell proliferation ability was evaluated using CCK-8 solution as described above.

### Colony formation assay

Cells were seeded into 12-well plates at a low density of 100 cells per plate and cultured for 14 days. The cells were then fixed with 95% methanol and pigmented with 0.1% crystal violet. The number of colonies with >10 cells observed under low magnification microscope were counted.

### Flow cytometry analysis of cell apoptosis

For the cell apoptosis analysis, PC3 and DU145 cells, 48 hour after transfection with miR-4638 mimic, miR-4638 inhibitor, si*Kidins220* and the scramble RNA negative controls (RIBOBIO), were collected and resuspended to 1 × 10^5^ cells in 195 μL of buffer. Then 5 μl Annexin V-FITC and 10 μl PI staining (Beyotime, Shanghai, China) were added into the cell mix and incubated for 15 minutes in the dark at room temperature. The apoptosis rates were determined using the flow cytometry-BD FACS Calibur (BD Biosciences) and Flowjo software one hour after staining.

### Matrigel tube formation assay

Microtubule formation assay was performed as previously described [81]. Briefly, 96-well plates were coated with 60 μl Matrigel solution (BD Bioscience, New Bedford, MA, USA) diluted at 1:1 in cell culture medium at 4°C. The plate was allowed to solidify for one hour at 37°C before cell seeding. The conditioned medium was collected from supernatant fluid of 48-hour cultured PCa cells with different transfections and filtered using 0.45 μm filter. HUVECs in 150 μl conditioned medium diluted at 2:1 in EBM-2 culture media without FBS, were seeded at density of 1 × 10^4^ cells per well. After incubation at 37°C for 6 hours, four randomly selected fields of each well were photographed. The angiogenesis index was calculated using the formula as previously described [81].

### Vasculogenic mimicry analysis

96-well plates were coated with 60 μl Matrigel solution diluted as a 1:1 mixture of High Concentration Matrigel (BD Bioscience) and culture medium without FBS, penicillin and streptomycin solution at 4°C. The plate was allowed to polymerize for one hour at 37°C. PC3 cells, stably transfected with pCDH-miR-4638-5p, pCDH-miR-4638-5p sponge, pCDH*-*sh*Kidins220*, pCDH*-*sh*AKT* and pCDH-vector controls in RPMI 1640 medium without FBS, were seeded at density of 1.5 × 10^4^ per well. After incubation at 37°C for 6 hours, three randomly selected fields of each well were photographed. The vasculogenic mimicry index was calculated using the same method as microtubule formation assay described above.

### Tumorigenicity analysis and Matrigel plug assay for angiogenesis analysis in nude mice

BALB/C nu/nu male athymic mice (3-4 weeks old) were purchased from Model Animal Research Center of Nanjing University (Nanjing, China). All animal experiments were evaluated and approved by the institutional ethics review board of National Institute of Health Guide for the Care and Use of Laboratory Animals and Wuxi No. 2 People's Hospital. The treated cells (PC3 cells infected with lentivirus) were harvested at a sub-confluent density, washed with phosphate-buffered saline (PBS) and re-suspended in serum-free medium. 2 × 10^7^cells in 500 μl of serum-free medium were mixed with 500 μl of High Concentration Matrigel (BD Biosciences) and 100 μl mixture (2 × 10^6^ cells) was immediately injected subcutaneously into the left or right flanks of nude mice. The tumor size was measured every 4 days with a two-dimensional caliper and tumor volume was calculated using the formula: 0.52 × length × width^2^. At day 28 after injection, mice were sacrificed and the xenograft tumors were surgically removed. Tumor samples were collected to make fresh frozen as well as formalin-fixed and paraffin-embedded sections for gene expression analysis. The hemoglobin content of the Matrigel plug was determined using Drabkin's reagent kit according to the manufacturer's instruction (Sigma-Aldrich) for tumor angiogenesis analysis. The final hemoglobin level was determined by spectrophotometric analysis at 540 nm.

### Immunohistochemistry staining

Formalin-fixed paraffin-embedded tissue samples from nude mice were immunostained using ABC method with antibodies against VEGF (Cat No. sc-152, 1:50 dilution) (Santa Cruz, CA, USA) and Ki-67 (Cat No. ab15580, 1:100 dilution) (Abcam, Cambridge, UK) according to the manufacturer's instructions as previously described [82].

### Luciferase reporter assay

Human *Kidins220* 3′-UTR sequence was obtained from NCBI gene database [83] and synthesized by Invitrogen. The full length *Kidins220* 3′-UTR sequence was amplified and cloned into pGL3-Control downstream of the luciferase gene to generate the plasmid pGL3-Luc-*Kidins220*-3′-UTR WT (*Kidins2*20-3′-UTR WT). Meanwhile, pGL3-Luc-*Kidins220*-3′-UTR Mut (*Kidins220*-3′-UTR mut) was generated from *Kidins220*-3′-UTR WT by changing three base pairs of the miR- 4638-5p seed-sequence. The wild-type or mutant reporter plasmids were co-transfected with the miR-4638- 5p or negative control and renilla vector *pRL- TK* into HEK293T cells (about 1 × 10^5^) at 50%–60% confluence using Lipofectamine 2000 reagent (Invitrogen) according to the manufacturer's instructions. After 48 hours, relative luciferase activity was measured using the Promega Dual-Luciferase Reporter (DLR) Assay System. At least three independent transfections were done for each experiment condition.

### Western blot analysis

Proteins were separated by 8% and 10% SDS polyacrylamide gels and electrophoretically transferred onto polyvinylidene fluoride membranes (Millipore, Billerica, MA, USA). The membranes were blocked overnight with 5% non-fat dried milk and incubated overnight with antibodies. Anti-Kidins220 rabbit polyclonal antibody (PAb) (Cat No. ab97345) was purchased from Abcam Inc (Abcam, Cambridge, UK). Anti-PI3K rabbit monoclonal antibody (MAb) (Cat No. ♯4257), anti-phospho-PI3K rabbit PAb (Cat No. ♯4228), anti-AKT rabbit PAb (Cat No. ♯9272) and anti-phospho-AKT (Cat No. ♯4060) were all purchased from Cell Signaling Technologies (Beverly, MA, USA). Anti-VEGF rabbit PAb (Cat No. sc-152), anti-GAPDH mouse MAb (Cat No. sc-47724), anti-a-Tubulin mouse MAb (Cat No. sc-23948) and horseradish peroxidase (HRP) conjugated goat anti-rabbit (Cat No. sc-2004) or anti-mouse (Cat No. sc-2005) secondary antibodies were obtained from Santa Cruz Biotechnology (Santa Cruz, CA, USA). The signals were detected by enhanced chemiluminescence (Millipore).

### Statistical analysis

All experiments were performed at least in triplicates unless otherwise mentioned. Numerical data were expressed as mean ± SD. Two group comparisons were analyzed by two-sided Student's *t*-test. *P* < 0.05 was considered significant.

## SUPPLEMENTARY MATERIALS


