# Supplementary material for: MiR-4638-5p inhibits castration resistance of prostate cancer through repressing Kidins220 expression and PI3K/AKT pathway activity
Source: Oncotarget. 2016 Jun 18;7(30):47444–64. doi: 10.18632/oncotarget.10165 (PMC5216953; doi:10.18632/oncotarget.10165)
Supplement: Supplementary file 1 [file oncotarget-07-47444-s001.pdf]

MiR-4638-5p inhibits castration resistance of prostate cancer through repressing Kidins220 expression and PI3K/AKT pathway activity

Supplementary Materials

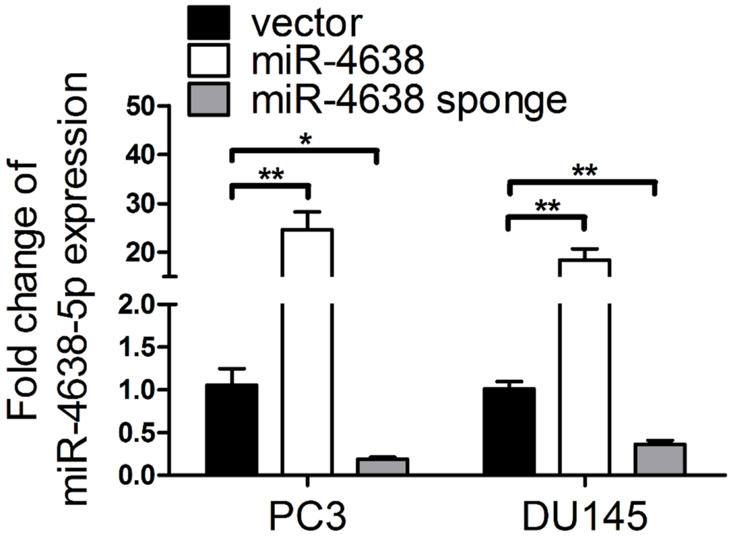

Supplementary Figure S1: miR-4638-5p expression were increased or decreased in PCa cells transduced by lentiviral miR-4638-5p and miR-4638-5p sponge respectively. miR-4638-5p expression in PC3 and DU145 cells transduced by lentiviral miR-4638-5p and miR-4638-5p sponge constructs were determined by qRT-PCR.

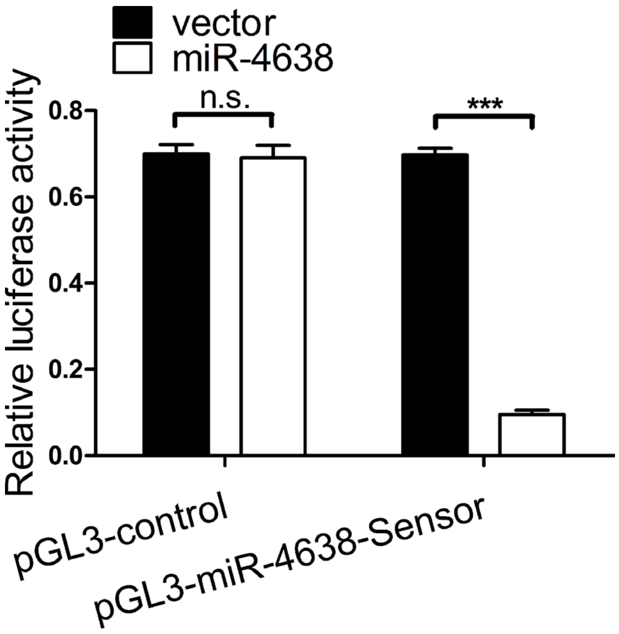

Supplementary Figure S2: miR-4638-5p inhibited the reporter activity of pGL3-miR-4638-5p sensor reporter. Luciferase activity was detected in lentivirus empty vector (vector) or lentivirus-miR-4638-5p (miR-4638) transduced HEK293T cells transfected by the pGL3-Control or the pGL3-miR-4638-5p sensor reporter (pGL3-miR-4638-Sensor).

Supplementary Table S1: Clinicopathological characteristics related to each PCa sample analyzed in this study

Patients	Age (years)	Clinical stage	Gleason score	Total PSA at diagnosis (ng/ml)	Free PSA at diagnosis (ng/ml)
ADPC1	52	T1	6	6.51	1.06
ADPC2	59	T1	6	10.59	1.38
ADPC3	63	T1	6	8.35	1.73
ADPC4	68	T2	7	13.12	0.46
ADPC5	65	T2	6	4.26	0.55
ADPC6	57	T2	6	16.47	1.75
ADPC7a	72	T2	6	6.76	1.04
ADPC8a	65	T2	6	11.22	1.37
ADPC9a	64	T2	7	26.38	4.25
ADPC10	70	T2	6	3.45	0.52
ADPC11	61	T2	6	14.24	2.01
ADPC12	64	T2	6	19.45	1.47
ADPC13	59	T2	6	20.23	3.38
ADPC14	64	T2	7	13.75	0.44
ADPC15	67	T2	7	27.73	2.72
ADPC16	72	T2	8	64.75	4.15
ADPC17	75	T2	8	81.09	6.39
ADPC18	69	T2	7	42.72	4.03
ADPC19	58	T2	6	14.88	1.16
ADPC20	63	T2	6	4.57	0.42
ADPC21	70	T2	6	16.24	2.27
ADPC22	74	T2	7	60.66	5.35
ADPC23	73	T3	8	37.35	4.26
ADPC24	67	T3	7	31.57	2.34
ADPC25	71	T3	7	22.41	1.41
ADPC26	56	T3	8	108.19	7.46
ADPC27	72	T3	7	19.57	2.27
ADPC28	63	T3	6	21.88	1.63
ADPC29	70	T3	6	33.44	4.56
ADPC30	62	T3	6	14.38	1.12
CRPC1a	67	T3	8	8.25	0.933
CRPC2a	75	T3	9	21.88	2.23
CRPC3a	70	T3	9	32.34	2.88
CRPC4	68	T3	7	6.82	0.83
CRPC5	71	T3	8	16.33	3.09
CRPC6	73	T4	9	30.63	1.73
CRPC7	75	T4	10	53.12	4.11
CRPC8	68	T4	10	91.74	5.57
CRPC9	73	T4	9	18.38	2.42
CRPC10	70	T4	8	9.25	1.13
CRPC11	73	T4	9	37.22	2.87
CRPC12	76	T4	10	74.57	3.42
CRPC13	68	T4	10	42.11	3.21
CRPC14	72	T4	9	16.34	1.37

CRPC15	75	T4	9	33.89	0.89
CRPC16	74	T4	10	60.56	4.37
CRPC17	71	T4	9	17.78	2.1
CRPC18	76	T4	10	53.78	3.69

[a]indicate that the cases were selected for miRNA microarray analysis.

Supplementary Table S2: 30 down-regulated (> 2 folds) miRNAs in CRPCs

		Normalized expression level							
**MiRNAs**	**Fold change (CR vs. AD)**	**CR1**	**CR2**	**CR3**	**AD7**	**AD8**	**AD9**	**Mean of CR group**	**Mean of AD group**
miR-135a-5p	0.0880	0.4089	0.5097	0.5718	7.7133	4.9650	4.2635	0.4968	5.6473
miR-1-3p	0.1207	0.0818	0.2464	0.0389	0.8284	1.0234	1.1901	0.1224	1.0140
miR-143-3p	0.0408	0.6893	4.1522	1.3017	60.619	56.390	33.577	2.0477	50.195
miR-145-3p	0.2430	0.3645	0.4155	0.2117	1.6095	0.9860	1.4860	0.3305	1.3605
miR-205-5p	0.0325	0.5631	0.7874	0.5158	29.447	18.687	9.3564	0.6221	19.163
miR-24-1-5p	0.2139	0.1168	0.2005	0.1119	0.7923	0.5467	0.6674	0.1431	0.6688
miR-4638-5p	0.4225	0.4346	0.6087	0.7737	2.0090	1.5958	0.6955	0.6057	1.4334
miR-23b-3p	0.0316	0.4112	0.3502	1.3674	22.964	28.563	15.866	0.7096	22.464
miR-27b-3p	0.0366	0.1402	0.1474	0.4550	6.6298	7.3505	6.3110	0.2475	6.7638
let-7b-5p	0.1986	5.7640	0.5990	5.1022	18.813	28.731	10.175	3.8218	19.240
miR-34a-5p	0.0992	0.5304	0.5483	0.6545	8.8104	5.0304	3.6307	0.5777	5.8238
miR-146a-5p	0.1679	0.0280	0.1087	0.0608	0.6366	0.4042	0.2549	0.0659	0.4319
miR-99a-5p	0.2316	1.1776	1.6957	0.7956	5.3567	7.1612	3.3218	1.2230	5.2800
let-7c-5p	0.1679	0.4907	0.4130	0.4769	2.4041	3.8061	2.0130	0.4602	2.7410
miR-342-3p	0.2650	0.8481	0.1546	0.2238	1.7675	1.3154	1.5464	0.4089	1.5431
miR-30a-5p	0.0406	0.1706	0.1836	0.7713	7.8939	10.888	8.9698	0.3751	9.2505
miR-30b-5p	0.1234	1.3972	1.6570	4.9319	26.158	24.054	14.311	2.6620	21.508
miR-101-3p	0.1856	11.706	4.4565	3.2068	45.212	30.320	28.808	6.4563	34.780
miR-16-5p	0.1803	2.5023	0.6304	2.3382	12.438	13.210	4.7019	1.8237	10.117
miR-15a-5p	0.1338	2.7523	0.3841	2.0365	14.977	15.757	7.9331	1.7243	12.889
miR-4288	0.4627	2.4463	2.4734	2.3552	4.4808	4.4065	6.8359	2.4250	5.2411
miR-338-3p	0.2084	0.0678	0.2343	0.1095	0.6659	0.6308	0.6782	0.1372	0.6583
miR-335-5p	0.1185	0.3061	0.2053	0.2409	2.6862	1.9393	1.7235	0.2508	2.1163
miR-4695-3p	0.3770	6.8738	5.8068	5.3942	17.932	16.215	13.793	6.0249	15.980
miR-378d	0.3362	0.1916	0.1522	0.3187	0.8713	0.7383	0.3607	0.2208	0.6568
miR-378c	0.2641	0.0865	0.1159	0.2190	0.3612	0.6425	0.5918	0.1405	0.5318
miR-3607-3p	0.2696	8.0117	4.1836	1.7932	24.142	12.192	15.549	4.6628	17.294
miR-200c-3p	0.2012	9.0467	5.3841	13.392	51.825	55.556	30.918	9.2742	46.096
miR-100-5p	0.0725	0.0491	0.1667	0.2579	1.8781	2.6168	2.0367	0.1579	2.1772
miR-30c-5p	0.2448	11.315	7.3720	3.3285	37.384	32.591	19.976	7.3386	29.984

Abbreviation: CR, CRPC and AD, ADPC.

Supplementary Table S3: 32 up-regulated (>2 folds) miRNAs in CRPCs

		Normalized expression level							
MiRNAs	Fold change (CR vs. AD)	CR1	CR2	CR3	AD7	AD8	AD9	Mean of CR group	Mean of AD group
miR-7-2-3p	4.1346	2.0047	2.0338	2.0900	0.5756	0.4790	0.4277	2.0428	0.4941
miR-9-3p	8.2878	1.0537	2.0845	1.0243	0.1196	0.1472	0.2354	1.3875	0.1674
miR-1247-5p	4.3514	0.9229	1.6932	0.2263	0.2032	0.1822	0.2678	0.9475	0.2177
miR-197-5p	3.3612	0.1262	0.1860	0.0681	0.0497	0.0397	0.0238	0.1268	0.0377
miR-663b	3.4999	1.0678	1.2560	0.3820	0.2754	0.2126	0.2851	0.9019	0.2577
miR-494-5p	3.4036	0.2921	0.3333	0.2336	0.1084	0.1051	0.0389	0.2863	0.0841
miR-592	2.4048	0.0794	0.1594	0.0170	0.0316	0.0467	0.0281	0.0853	0.0355
miR-150-5p	2.3261	1.6379	2.9275	1.0462	0.7765	0.8411	0.7948	1.8705	0.8042
miR-18a-3p	4.2836	0.0794	0.2681	0.1995	0.0248	0.0467	0.0562	0.1824	0.0426
miR-373-3p	4.6736	0.0608	0.3213	0.0414	0.0113	0.0491	0.0302	0.1411	0.0302
miR-520b/ 520c-3p	2.1076	0.2874	0.2826	0.1849	0.0926	0.1425	0.1231	0.2516	0.1194
miR-1307-3p	3.5505	0.1729	0.2295	0.1557	0.0406	0.0561	0.0605	0.1860	0.0524
miR-501-5p	4.5149	2.7897	1.843	4.4282	0.7156	0.6542	0.6372	3.0203	0.6690
miR-3652	2.1661	0.1519	0.2391	0.1922	0.0858	0.0841	0.0994	0.1944	0.0898
miR-4475	3.6778	4.0654	4.2391	3.0122	1.0429	0.9370	1.0972	3.7722	1.0257
miR-3074-5p	2.8092	0.0724	0.1594	0.1144	0.0294	0.0421	0.0518	0.1154	0.0411
miR-554	2.8476	0.7056	0.8889	1.0292	0.1693	0.3201	0.4320	0.8746	0.3071
miR-4653-3p	4.0617	3.5818	4.7053	3.3625	0.9142	1.0187	0.9352	3.8832	0.9560
miR-3136-3p	2.6238	6.8458	5.6256	3.2920	2.2145	1.7804	2.0130	5.2545	2.0026
miR-3913-5p	3.8982	0.1355	0.2802	0.1728	0.0384	0.0608	0.0518	0.1962	0.0503
miR-371a-3p	5.7032	0.3481	0.5918	0.2457	0.0564	0.0608	0.0907	0.3952	0.0693
miR-513a-5p	5.1661	7.4720	8.8285	6.6277	1.4560	1.3949	1.5875	7.6427	1.4794
miR-1251-3p	2.0544	1.1122	0.7826	1.0827	0.5373	0.5794	0.3326	0.9925	0.4831
miR-595	3.4638	0.3855	0.4686	0.6107	0.1332	0.1752	0.1145	0.4883	0.1410
miR-661	2.2673	0.1589	0.1957	0.1119	0.0564	0.0608	0.0886	0.1555	0.0686
miR-1976	2.5308	1.0351	1.3454	0.6399	0.3544	0.4136	0.4255	1.0068	0.3978
miR-522-3p	6.5087	0.5654	0.5580	0.2166	0.0677	0.0561	0.0821	0.4467	0.0686
miR-204-5p	3.9015	0.5257	0.3744	0.2895	0.0452	0.0935	0.1663	0.3965	0.1016
miR-411-3p	2.1986	0.6752	0.7101	0.6886	0.2483	0.1939	0.5011	0.6913	0.3144
miR-1471	10.710	0.4393	0.6232	0.5791	0.0339	0.0654	0.0540	0.5472	0.0511
miR-1207-3p	3.3984	0.6308	1.1208	0.8175	0.1783	0.1846	0.3931	0.8564	0.2520
miR-515-3p	4.7751	0.0654	0.1643	0.1363	0.0045	0.0397	0.0324	0.1220	0.0255

Abbreviation: CR, CRPC and AD, ADPC.
